# Helical‐Like Assembly of Nateglinide as Coating for Oral Delivery of Insulin and Their Synergistic Prevention of Diabetes Mellitus

**DOI:** 10.1002/advs.202301879

**Published:** 2023-08-16

**Authors:** Yanfei Li, Lihang Chen, Yu Xu, Sihui Li, Huijia Yan, Tao Chen, Ziqi Hua, Di Wu, Runan Zhao, Jiangning Hu

**Affiliations:** ^1^ SKL of Marine Food Processing & Safety Control National Engineering Research Center of Seafood Collaborative Innovation Center of Seafood Deep Processing School of Food Science and Technology Dalian Polytechnic University Dalian 116034 China

**Keywords:** diabetes, helical nanofibers, nateglinide, oral delivery, self‐assembly

## Abstract

Oral delivery of antidiabetic active components promises to free millions of people from daily suffering who require routine injections. However, oral insulin (Ins) and other short‐acting compounds such as nateglinide (NG) in harsh gastrointestinal tract still face great challenging, including low bioavailability, and rapid elimination. In this study, inspired by the self‐assembly of phenylalanine‐based peptides in nature, it is showed that NG a small phenylalanine derivative, assembles into left‐handed helical nanofibers in the presence of Ca^2+^. These helical NG nanofibers functioned as a coating layer on the surface of Ca^2+^‐linked alginate (Alg) microgels for the effective encapsulation of Ins. As expected, the sustained release and prolonged circulation of Ins and NG from the Ins‐loading Alg/NG microgels (Ins@Alg/NG) in the intestinal tract synergistically maintain a relatively normal blood glucose level in streptozotocin‐induced diabetic mice after oral administration of Ins@Alg/NG. This further confirms that Ins@Alg/NG ameliorated Ins resistance mainly through activating Insreceptor substrate 1 (IRS1), protein kinase B (AKT), and AMP‐activated protein kinase (AMPK), as well as by repressing glycogen synthase kinase‐3β (GSK‐3β). The strategy of using the assembly of NG as a coating achieves the oral delivery of insulin and showcases a potential for the treatment of diabetes.

## Graphical abstract

In brief, nalglinide (NG) can form a hydrogel coating on the surface alginate microspheres (Alg) rich in calcium ions, and encapsulate Ins in the microspheres to obtain Ins‐loading microspheres (Ins@Alg/NG). Through oral administration, NG and Ins are successfully released in the intestine to explore the synergistic protective effect and mechanism of the two on diabetic mice.

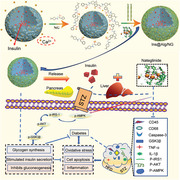

## 1 Introduction

Diabetes is chronic disease that affects human health and undermines the quality of life of patients. Over four hundred million people worldwide suffer annually from severe secondary complications caused by diabetes.^[^ ^1^, ^2^ ^]^ To control the blood glucose level in diabetic patients, various interventions, including insulin (Ins) therapy, are successfully developed and applied in clinical settings.^[^ ^3^, ^4^, ^5^, ^6^, ^7^ ^]^ Oral Ins treatment is a promising strategy that could potentially relieve millions of people from the daily discomfort of subcutaneous Ins injections.^[^ ^8^, ^9^, ^10^ ^]^ The convenience of oral Ins may encourage earlier initiation of Ins therapy, improve patient compliance, and ultimately lead to better glucose control and long‐term outcomes.^[^ ^11^ ^]^ Nevertheless, being similar to many protein components, oral Ins still faces significant challenges such as poor gastrointestinal stability,^[^ ^12^, ^13^, ^14^ ^]^ rapid metabolism,^[^ ^15^, ^16^, ^17^ ^]^ quick systemic elimination, and both easy aggregation and difficult absorption face great challenges.^[^ ^18^, ^19^, ^20^ ^]^ Which leads to low therapeutic efficiency. As a result, there are currently only a few commercial oral Ins formulas available for use in clinics. Fortunately, Ins delivery by microgels and nanomaterials has attracted much attention, the research shows that neonatal Fc receptor‐targeted lignin‐encapsulated porous silicon nanoparticles for enhanced cellular interactions and Ins permeation across the intestinal epithelium,^[^ ^21^ ^]^ peptide molecules (Ins) was formulated into nano‐particles and their surface is decorated with carrier polymers, consequently, realizes ultrahigh peptide loading degree (up to 78.9%).^[^ ^22^ ^]^ These fully indicate that nano‐carriers have a good application prospect in Ins delivery.

To better control the blood glucose levels in daily life, many auxiliary oral components are suggested alongside with the Ins treatment.^[^ ^23^, ^24^, ^25^, ^26^ ^]^ Among these, nateglinide (NG), a non‐sulfonylurea class of antidiabetic compounds, is considered an important oral Ins secretagogue, which can bind ATP receptors of pancreatic β‐cells, close potassium channels, and activate calcium channels to effectively stimulate Ins secretion in β‐cells and promote Ins secretion, thus achieving the uptake of excess glucose from the blood and a decrease in hyperglycemic levels.^[^ ^27^, ^28^ ^]^ Many reports reveal that NG can effectively control the postprandial blood glucose level in early type 2 diabetic patients.^[^ ^29^, ^30^, ^31^, ^32^ ^]^ According to a five‐year double‐blind clinical trial, it was reported that NG used as a short‐term effector did not effectively reduce the incidence of diabetes when used as the sole treatment drug.^[^ ^33^ ^]^ The administration of NG at the wrong time may increase the risk of blood glucose fluctuations and sudden hypoglycemia, due to its rapid absorption and elimination in vivo, as supported by studies.^[^ ^34^, ^35^ ^]^ To address this issue, developing efficient delivery systems for oral NG could be the best approach to prolong its blood circulation and improve its short‐term pharmacokinetics in vivo.^[^ ^36^ ^]^ It is worth noting that NG is a D‐phenylalanine derivative, and recent studies have shown that phenylalanine‐based peptides have a unique tendency to self‐assemble.^[^ ^37^, ^38^ ^]^ In numerous groundbreaking studies, the emphasis was on the stacking interaction of diphenylalanine in forming well‐organized nanostructures like nanofibers, nanovesicles, nanowires and nanotubes, as evidenced by several reports.^[^ ^39^, ^40^, ^41^, ^42^ ^]^ Additionally, it has been observed that other aromatic amino acids such as tyrosine‐rich short peptide, can also assemble into macroscopic 2D nanosheets.^[^ ^43^ ^]^ Inspired by the extensive studies on self‐assembly of diphenylalanine and other aromatic amino acid‐rich peptides, we hypothesize that NG, a derivative of phenylalanine, may also exhibit a tendency for self‐assembly despite not being a peptide. The resulting assembled form of NG could have a wide range of applications and potentially increase its effectiveness in vivo.

With these purposes, we first examined the self‐assembly capability of NG. Unfortunately, NG did not form any aggregations but a clear solution under the condition of the self‐assembly of diphenylalanine peptide as previously reported.^[^ ^44^ ^]^ Serendipitously, when small amount of cation calcium Ca^2+^ was added into the solution, a milky gel was surprisingly formed. This phenomenon differed from the self‐assembly of diphenylalanine peptide and other oligopeptides, likely due to the cyclohexane moiety on the backbone of NG impeding self‐assembly behavior, while cations triggered the assembly. More interestingly, under scanning electron microscopy (SEM) and atomic force microscopy (AFM) revealed fine nanofibers with left‐handed helices in the NG gel. The study prompted us to conduct further research on the assembly mechanism of NG using both all‐atom and coarse‐grained molecular dynamics simulations. Clearly, the stabilization of helical assembly of NG primarily due to ionic bonds between the carboxylate anion of NG and Ca^2+^, as well as π–π interactions between the benzene rings of NG within a distance range of 4.8 and 3.7 Å, and intermolecular hydrogen bonds with H···O distances of 2.5 Å also played a role. These helical nanofibers were found to be resistant to degradation in gastric acid and achieved long‐term release in the intestinal tract.

Considering these excellent characteristics of the gel and the role of Ca^2+^ in the gel formation, the helical assembly of NG was expected to form coating on the surface of Ca^2+^‐linked alginate microgel template. This coating would then encapsulate the Ins, effectively protecting it from degradation and inactivation in the harsh gastrointestinal environment. Meanwhile, the sustained release and prolonged circulation of Ins and NG from Ins‐loading alginate/nateglinide microgels (Ins@Alg/NG) in intestinal tract was demonstrated to synergistically maintain the relatively normal blood glucose level and restore the functions of damaged pancreatic islets in streptozotocin (STZ)‐induced diabetic mice after oral administration once for every 3 days.

## 2 Results and Discussion

### 2.1 Characterization of Helical‐Like Nanofibers from Nateglinide

**Figure 1:**
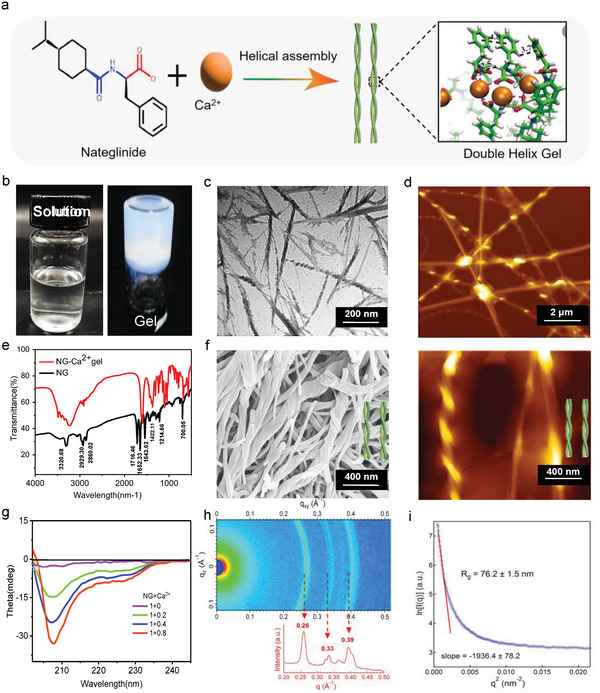
Characterization of NG‐Ca2+ gel. a) schematic illustration of helical assembly of NG. b) NG‐Ca^2+^ gel. c,d,f) TEM, AFM, and SEM images of NG‐Ca^2+^ gel. e) FT‐IR spectra of NG and NG‐Ca^2+^ gel. g) CD spectra of NG‐Ca^2+^ gel. h) 2D‐WAXS profiles of the NG‐Ca^2+^ gel. i) The Guinier plot for the NG‐Ca^2+^ gel.

**Figure** **1**a illustrates the schematic illustration of NG self‐assembly. To examine the gelling behavior of NG, we dissolved it in phosphate buffer at pH 9 up to a concentration of 30 mg mL^−1^ using heating and cooling processing. The result was a clear liquid solution as shown in Figure 1b. Upon addition of Ca^2+^ ions to the NG solution, a milky gel with the ability to be placed vertically was observed. This phenomenon intrigued us to further observe the morphology of the NG gel using various techniques such as transmission electron microscopy (TEM), atomic force microscopy (AFM), and scanning electron microscopy (SEM). As evidenced by Figure 1c,d,f, the NG gel consisted of densely intertwined nanofiber networks. The fibers, which exhibited left‐handed helices with an average diameter of ≈200 nm, were observed through 3D scanning of the AFM (refer to Figure S1a in the Supporting Information). Circular dichroism (CD) spectroscopy was utilized to demonstrate the presence of helical structures in the gel (Figure 1g). The intensity of the Cotton effect, which was characterized by negative peaks at 208 and 222 nm (typical signals of α helix structure), greatly increased and amplified with increasing Ca^2+^ concentrations from 0.2 to 0.8 mg, this confirms that Ca^2+^ triggered the chiral assembly of NG.

The structural evolution of NG during helical assembly was analyzed using Fourier Transform Infrared (FT‐IR) spectroscopy and Small Angle X‐ray Scattering (SAXS) techniques. The FT‐IR spectra (Figure 1e) illustrated that the peak of NG at 1716.46 cm^−1^, which was assigned to the C = O stretching vibration of hydrogen bonded carboxylic acids, disappeared completely after the assembly. Meanwhile, other peaks at 1214.66 cm^−1^ for the stretching vibration of C─N bond of NG amide, 3320.68 and 1652.33 cm^−1^ for the stretching vibration of N─H bond and C═O double bond of amide, respectively, exhibited a blue‐shift, indicating the possible existence of hydrogen bonding during the assembly process. The gel exhibited wider peaks ranging from 3320–2860 cm^−1^, indicating that hydrogen bonding was the primary driving force during assembly. The crystalline properties of the NG gel were confirmed using SAXS, which showed clear scattering peaks at 0.26, 0.33 and 0.39 q (Å)^−1^, corresponding to crystallite spacing at 24.15, 19.03, and 16.10 nm (Figure 1h). Additionally, the rotation radius of the NG gel was calculated as 76.2±1.5 nm, which was similar to the diameter indicated by AFM and SEM images (Figure 1i). To test the acid‐resistance of the NG gel, its morphology was observed using SEM after 3 and 6 h in a strong acidic solution (pH 1) (Figure S1b, Supporting Information). The NG fiber networks remained intact and exhibited exceptional acid resistance properties. Additionally, we analyzed the mechanical properties of the gel using oscillatory shear rheology (Figure S2a,b, Supporting Information). When the strain exceeded 75% or temperature scanning was over 53 °C, the loss modulus (G″) was higher than the storage modulus (G″), indicating a transition from gel to solution (solution state: G″ > G″, gel state: G' < G“). Rotational rheological measurements were performed to examine viscoelastic properties of NG gel. Dynamic frequency scans also showed that the value of G' was much higher than that of G” (Figure S2c, Supporting Information), indicating that NG gel possessed good mechanical properties.

Experimental tests have shown that the assembly process was influenced by concentrations, pH values, and cation types. The minimum gelation concentration of NG was found to be 25 mg mL^−1^, with increasing concentration leading to oversaturation and aggregations (Figure S3a, Supporting Information). Additionally, an optimal pH value of 9 was identified, which was capable of dissolving NG into a clear solution and forming a gel after the addition of Ca^2+^ (Figure S3, Supporting Information). To investigate the impact of various cations on the formation of NG gel, monovalent cations (K^+^, Na^+^ and Ag^+^), divalent cations (Zn^2+^ and Mg^2+^) and trivalent cations (Fe^3+^ and Al^3+^) were introduced into the NG solution instead of Ca^2+^(as shown in Figure S3c, Supporting Information). Only Zn^2+^ and Mg^2+^ were found to trigger the assembly of NG and form gel, which was also observed by TEM and SEM images (as depicted in Figure S4, Supporting Information). Interestingly, the NG gel network fibers supplemented with Ca^2+^ exhibited a helical shape under the same conditions. However, no comparable network fibers were observed in the NG gel that was supplemented with Zn^2+^ and Mg^2+^.

### 2.2 Molecular Dynamics Simulation of Helical Assembly of Nateglinides

**Figure 2:**
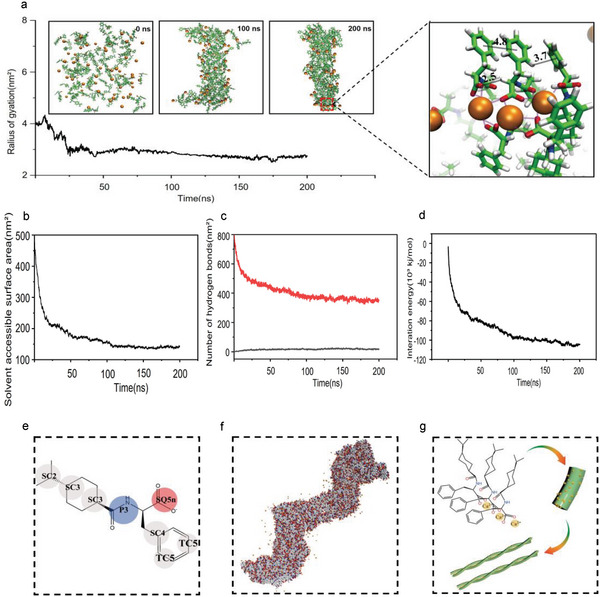
Molecular dynamics simulation of helical assembly of NG. a) Deprotonated NG and Ca^2+^ were dispersed in the system. b) The solvent accessible surface area. c) the number of NG‐water hydrogen bonds. d) Electrostatic interaction energy between deprotonated NG and Ca^2+^. e) coarse‐grained (CG) simulations using the Martini 3 force field. f) Deprotonated NG and Ca^2+^ were assembled into a helical segment. g) A larger helical segment by ionic bonding of their respective carboxylate anion and Ca^2+^.

All‐atom molecular dynamics (MD) simulations using Amber 14 sb (parmbsc1) force field were applied to explore the assembly mechanism of NG triggered by Ca^2+^.^[^ ^45^ ^]^ The simulation captured snapshots of the dynamic formation and evolution of the radius of gyration (R_gyr_) the solvent‐accessible surface area (SASA) of the NG nanofibers system. Initially, the deprotonated NG and Ca^2+^ were dispersed throughout the system, as illustrated in **Figure** **2**a. After being equilibrated through molecular dynamics simulations for a duration of 200 nanoseconds at a constant temperature of 298 K, the configuration displayed the dynamic assembly of deprotonated NG and Ca^2+^ into a helical‐like cluster. This cluster had a radius of gyration of ≈2.7 nanometers, as shown in Figure S5a–c and Movie S1, (Supporting Information). Moreover, the stabilization of the cluster was dominated by ionic bonds between the carboxylate anion on NG and Ca^2+^, π–π interactions between benzene rings on NG ranging from 4.8 and 3.7 Å, and intermolecular hydrogen bonds with H···O distances at 2.5 Å. The study shows that as the simulation time increased, solvent accessible surface area (SASA), the number of NG‐water hydrogen bonds and electrostatic interaction energy between deprotonated NG and Ca^2+^ decreased. This supports the idea that hydrophobic interaction and electrostatic force played a role in creating a favorable conformation during the self‐assembly process, as depicted in Figure 2b–d.

To gain more insight into longer timescales and larger length scales, we conducted coarse‐grained (CG) simulations utilizing the Martini 3 force field. The topology for deprotonated NG was established using Alessandri, R. et al.’s 2021 work (Figure 2e) as a reference.^[^ ^46^ ^]^ In particular, the branched alkane, secondary amide and carboxylate anion on the NG backbone were assigned to the SC2, P3 and SQ5n bead, respectively. Cyclohexane was represented by two SC3 beads, while methylbenzene was assigned an SC4 and two TC5 beads. The results showed that the average SASA of coarse‐grained MD was ≈3.4% smaller than the All‐atom MD, indicating the acceptability of the system (Figure S5d, Supporting Information). After equilibration for 1 µs at a constant temperature of 298 K, deprotonated NG and Ca^2+^ were assembled into a helical segment with a diameter of 7±2 Å, as expected (Figure 2f). In addition to the helical structure, the CG simulation revealed new insights into the mechanism by which the nano helical segments were assembled. The simulation demonstrated that cyclic hydrocarbon of deprotonated NG tended to gather in the inner part of the structure under hydrophobic interaction, while carboxylate anion of deprotonated NG and Ca^2+^ were exposed to water. Moreover, in certain cases, two small fragments were fused together to form a larger helical segment through ionic bonding of their respective carboxylate anion and Ca^2+^ (Figure 2g).

### 2.3 Nateglinide Nanofibers as Coating for Encapsulation of Insulin

**Figure 3:**
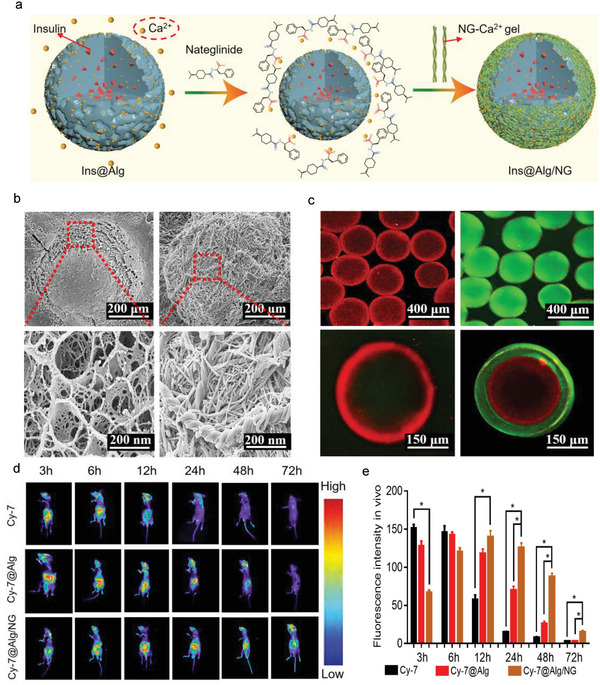
Characterization and distribution of Ins@Alg/NG in vivo. a) Diagram of NG formation. b) SEM of Ins@Alg and Ins@Alg/NG. c) The core‐shell morphologies of Ins@Alg/NG were observed by laser confocal microscopy (Alg was stained in red with rhodamine b and NG was stained in green with fluorescein isothiocyanate). d,e) Fluorescent distribution and intensity of free Cy7, Alg@Cy7, and Cy7@Alg/NG in vivo at different time points. Quantification of fluorescence intensity. Data were presented as mean ± s.e.m (n = 3). Statistical significance was assessed using one‐way ANOVA with Bonferroni's post‐test. *p < 0.05 was recognized as significant.

Inspired by the phenomenon of the assembly of NG nanofibers triggered by Ca^2+^, we chose Ca^2+^‐crosslinked alginate (Alg) microgels as template to form NG coating on the surface of Alg microgels since there were plenty of Ca^2+^ on the surface of microgels. These alginate/nateglinide (Alg/NG) core shell networks were designed to facilitate the oral delivery of Ins. The acid‐resistant NG coating prevented Ins degradation in the harsh gastrointestinal tract and also helped regulate blood glucose level during the gradual release of Ins and NG from the Alg/NG microgels in the intestinal tract (as shown in **Figure** **3**a). The successful construction of Alg/NG microgels was further demonstrated by the change in zeta potential from its native value to a positive value, as shown in Figure S6 in the (Supporting Information). SEM images (Figure 3b) revealed that the surface of Alg microgel, which had coarse porosities, was covered with a layer of coating as the particle size increased from 352.99 to 377.3 µm (Figure S7a,b, Supporting Information). This indicates that NG was successfully assembled on the surface of Alg microgels, as expected. This figure compares an Alg microsphere without NG coating and an Alg/NG microsphere with the NG coating (Figure S7c, Supporting Information). To further visualize the core/shell microstructures, rhodamine b and fluorescein isothiocyanate were used to stain Alg and NG, respectively. Under laser confocal microscopy, the red fluorescent Alg microgels were observed to be clearly covered with blue fluorescent NG coating, as depicted in Figure 3c. Ins was encapsulated in Ins@Alg/NG microgels, resulting in a final payload of 10%. The encapsulation rate was calculated to be 83.5%. The loading capacity and encapsulation rate of NG in Ins@Alg were 13.5% and 77.2%, respectively.

### 2.4 Stability and Distribution of Ins@Alg/NG in Gastrointestinal Tract

To investigate the stability of Ins‐loading Alg/NG microgels (Ins@Alg/NG) in gastrointestinal conditions, the microgels were exposed to various simulated digestion fluids for up to 48 h. Changes in morphology were visually observed at different time intervals were observed visually by microscopic imaging. As demonstrated in Figure S8a of the (Supporting Information), Ins@Alg/NG remained morphologically unaltered in gastric fluid even after 24 h, which suggests that it was able to withstand the gastric environment. While there was no significant change in Ins@Alg/NG morphology in intestinal fluid within 6 h, after 12 h, the microgels appeared to swell noticeably. Ins@Alg/NG microgels were observed to swell and develop numerous cracks after 24 h, and subsequently exploded after 48 h. This indicates that these microgels underwent gradual degradation and released Ins and NG in colonic fluid. The accumulative release of Ins and NG from Ins@Alg/NG microgels was further studied. As shown in (Figure S8b, Supporting Information), the release of Ins in colon fluid reached over 90% after 24 h, compared with these in intestinal fluid and gastric fluid (64.3% and 21.4%, respectively). The similar trend was also found in the release of NG from Ins@Alg/NG microgels (Figure S8c, Supporting Information). The results suggest that the Ins@Alg/NG microgels, which were resistant to gastric acid, possessed the ability to sustain the release of both Ins and NG in vivo. To further validate the successful formation of the NG coating on the Alg microsphere surface, we observed the changes in the color of the Alg microspheres from colorless to white after the NG coating, providing visual confirmation of successful NG coating formation on Alg microspheres (as shown in Figure S8d, Supporting Information).

Further, biological distributions of Ins@Alg/NG microgels in vivo were explored using a small animal living fluorescent imaging. Instead of Ins, Cy7 was used as a fluorescent probe in the microgels. The results showed that after oral administration, Alg/NG microgel‐treated mice had an intensified fluorescent signal and long‐time retention in the intestinal tract and whole body for up to 48 h. In contrast, free Cy7‐treated mice had a weak signal that disappeared after 12 h (Figure 3d; Figure S9, Supporting Information), indicating that Alg/NG microgels achieved prolonged retention of drugs in the gastrointestinal tract.

### 2.5 Efficacy of Ins@Alg/NG in STZ‐Induced Obese Diabetic Mice

**Figure 4:**
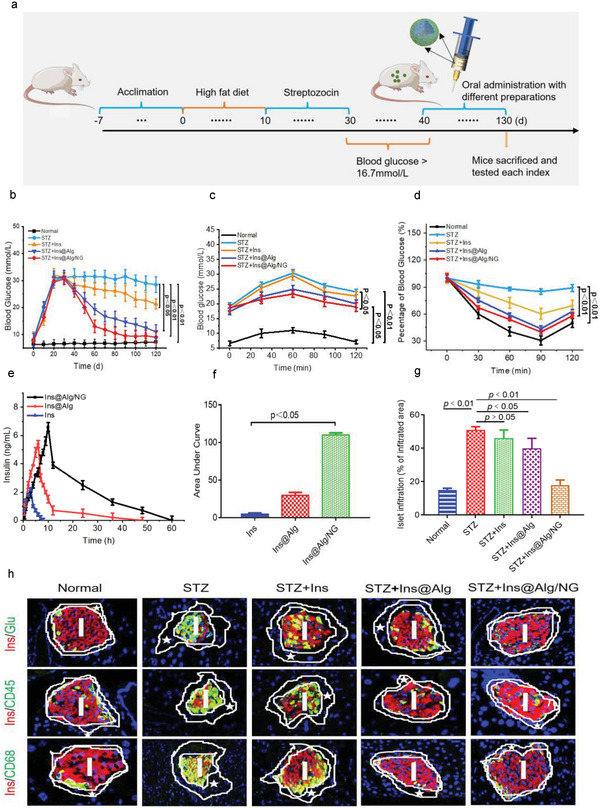
Ins@Alg/NG decreased the blood glucose level in diabetic mice by enhancing pancreatic function. a) Diagram of feeding and experimental process of diabetic mice. b) Blood glucose levels of mice was monitored by different drugs at different time intervals. c) Oral glucose tolerance test (OGTTs) was used to assess individual glucose tolerance. Fasting mice were given by 2 g glucose kg^−1^ body weight by gavage, and blood glucose levels were measured at 0, 30, 60, 90, and 120 min. d) Ins tolerance test (IPITTs) was used to assess individual Ins tolerance. Mice were rapidly injected with 2 g glucose kg^−1^ body weight (bw), followed by injection withIns (2 IU kg^−1^) body weight (bw), and blood glucose levels were measured at 0, 30, 60, 90, and 120 min. e) In vivo pharmacokinetics of insulin. f) Area under curve (AUC) of random Ins levels. g) Effects of Ins@Alg/NG on pancreatic islet morphology and immune cell infiltration. h) Immunostaining of mouse islets with Ins (red) and glucagon (green; top 1 rows); Ins (red) and CD45 (green; second row); Ins (red) and CD68 (green; bottom row). Data were presented as mean ± s.e.m (n = 7‐10). Statistical significance was assessed using one‐way ANOVA with Bonferroni's post‐test. p < 0.05 or 0.01 was recognized as significant.

To evaluate the effectiveness of Ins@Alg/NG in regulating hypoglycemia in vivo, we created a model of obese diabetic mice induced by a high‐fat diet and streptozocin (STZ). We monitored the random blood glucose level (BGL) using a blood glucometer for a total of 120 days. We considered mice with BGL levels ≥16.7 mmol L^−1^ lasting for 10 days as diabetic mice. After hyperglycemic adaptation for 40 days, the mice were orally gavaged for treatment with Ins@Alg/NG, Ins@Alg (without NG coating), free Ins (Ins, 20 IUkg^−1^) every 3 day, respectively (**Figure** **4**a). As shown in Figure 4b, the BGLs in the STZ group maintained at very high level ≈ 30 mmol L^−1^. Significantly, Ins@Alg/NG treatment greatly decreased the BGLs and gradually reached to normal basic blood glucose level <16.7 mmol L^−1^ after 90 days. In contrast, the groups administered with free Ins and Ins@Alg demonstrated only a limited decrease in blood glucose levels, with reductions of 25 and 16.7 mmol L^−1^, respectively, over a 120‐day period in diabetic mice. These findings indicate that the continuous release of Ins and NG from Ins@Alg/NG acted together to effectively lower blood glucose levels in diabetic mice. The symptoms observed in diabetic mice including weight loss, binge drinking and overeating were markedly improved (Figure S10a–c, Supporting Information). In order to fully illustrate the role of NG on the anti‐hyperglycemic effects and their synergistic effects between NG and Ins, we further conducted additional experiments anti‐hyperglycemic effects in diabetic mice for a duration of 80 days. Our data reveals that on the 80th day, the blood glucose level in mice receiving Alg/NG was 15.9 mmol L^−1^ (Figure S10d, Supporting Information), in contrast to a significantly lower 10.5 mmol L^−1^ observed in mice receiving Ins@Alg/NG under the same conditions. Additionally, histologic H&E, Masson, and immunofluorescence staining (Figure S10e, Supporting Information) indicated that Ins@Alg/NG demonstrated significantly better organ protective effects compared to Alg/NG alone. Our results suggest that the observed hypoglycemic function might result from a synergistic effect between Ins and NG, rather than the effect of NG alone. We then assessed the anti‐Ins resistance efficiency using the oral glucose tolerance tests (OGTTs) and intra‐peritoneal Ins tolerant tests (IPITTs) of mice after 120‐day oral administration of Ins@Alg/NG. The mice were fasted overnight before receiving an oral glucose treatment of 2 g kg^−1^ bw. Blood glucose levels were measured at 0, 30, 60, 90, and 120 min after treatment. The results of the oral glucose tolerance test (OGTT) showed that Ins@Alg/NG significantly improved glucose metabolism, as demonstrated in Figure 4c. Meanwhile, the IPITT tests also showed a significant improvement in Ins sensitivity in diabetic mice after treated with Ins@Alg/NG (Figure 4d). To investigate the pharmacokinetic parameters of Ins@Alg/NG, we administered different dosage forms orally to mice and studied their serum Ins levels for 60 h (Figure 4e). The free Ins‐treated group had a lower total serum Ins level, indicating less effective absorption and serum Ins content was almost zero after 8 h. However, the Ins@Alg/NG‐treated group had a serum Ins level that lasted for ≈60 h. The area under the curve (AUC_0‐60 hours_) of the Ins@Alg/NG‐treated group demonstrated both efficient absorption and a beneficial slow‐release effect, as illustrated in Figure 4f.

### 2.6 Effects of Ins@Alg/NG on Pancreatic Islet Function

Pancreatic islet function is essential for maintaining blood glucose level. Pancreatic beta cells in endocrine islets are sole organ of Ins production in mammals.^[^ ^47^ ^]^ To further investigate the effects of Ins@Alg/NG on the protection of pancreatic islet in diabetic mice, pancreatic islet morphology and islet immune cell infiltration were specifically examined by immunofluorescent staining. As shown in Figure 4h (top row, islets were delineated by solid lines, islet area was marked as “I”, and infiltrated area was labelled as “*”), pancreatic sections were double stained with a combination of glucagon in green and Ins in red, the two major islet hormones responsible for maintaining the balance of blood glucose. As we all know, the islets of healthy mice are oval in shape with diameter between 50 and 200 µm. Around 80% of β‐cells were generally located in the core of the islet, while others were distributed in outer side of islet. Surprisingly (Figure 4g), the islets from Ins@Alg/NG‐treated mice showed the quite normal morphology and minimal inflammation cell infiltration (Normal group, 15±1%; STZ group, 51±2%; STZ+Ins group, 46±5%; Ins@Alg group, 40±6%; Ins@Alg/NG group, 18±3%), which were comparable to normal group (Figure S11a,b, Supporting Information), Ins@Alg/NG group also showed more uninfected islet area (3031±198 µm^2^ for Ins@Alg/NG group; 91.8% over Normal group; 211.2% over STZ group; 121.2% over STZ+Ins group; 109.7% over Ins@Alg group) and positive β‐cell area (part of red is positive β‐cell: 2868±132 µm^2^ for Ins@Alg/NG group; 94.3% over Normal group; 436.5% over STZ group; 416.9% over STZ+Ins group; 138.7% over Ins@Alg group). These results revealed that Ins@Alg/NG were capable of alleviating and restoring the damage of the pancreatic islets and β‐cells attacked by immune cells during the diabetic progression.

To further explore how immune cell infiltration encroached on pancreatic islets, pancreatic sections were double stained with Ins in red and the lymphocyte markers CD45 and CD68 in green (Figure 4h, second and third row). As expected, little green fluorescence for CD45 and CD68 was detected in islets of Ins@Alg/NG‐treated mice, further indicating that the minimal immune cell infiltration occurred in Ins@Alg/NG‐treated mice. In contrast, other groups especially in STZ‐induced diabetic mice and free Ins‐treated mice showed severe immune cell infiltration with intensified green signals. Also, immunocytochemistry staining (Figure 6a) of pancreatic tissue sections showed that the highest levels of Ins and lowest levels of glucagon were in Ins@Alg/NG‐treated mice, compared with other groups, which was consistent with the immunofluorescent results described above, the number of β‐positive cells increased and the number of α‐positive cells decreased (Figure 6b,c). These findings suggest that the pancreatic damage during the diabetic progression was severe. Ins@Alg/NG could potentially reverse the negative effects of STZ‐induced cell apoptosis and aid in the regeneration of pancreatic islets in diabetic mice.

### 2.7 Alleviation of Ins@Alg/NG on Diabetic Complications

**Figure 5:**
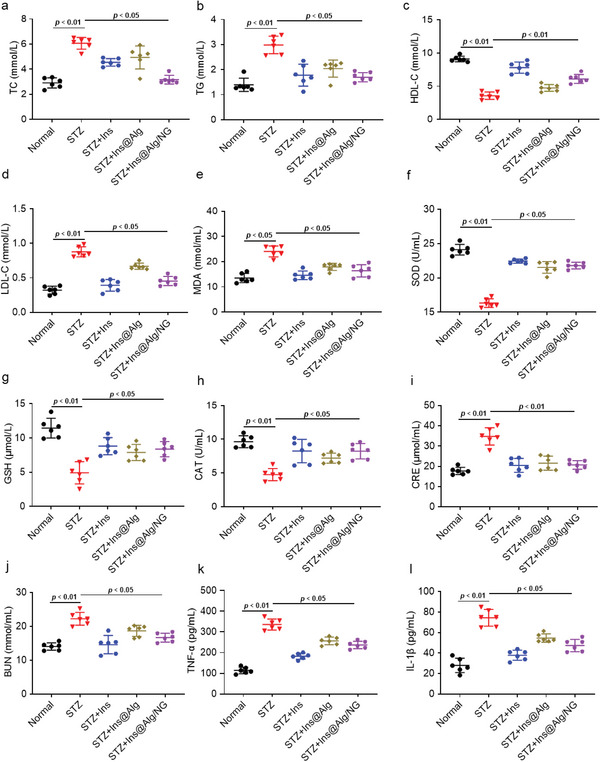
Ins@Alg/NG alleviated the diabetic complications. a–d) Four blood lipids including TC, TG, HDL‐C and LDL‐C. e–h) Four basic oxidation index including MDA, SOD, GSH, CAT. i,j) Renal function index CRE and BUN. k,l) Inflammation indicators TNF‐α and IL‐1β. Data were presented as mean ± s.e.m (n = 7–10).Statistical significance was assessed using one‐way ANOVA with Bonferroni's post‐test. p < 0.05 or 0.01 was recognized as significant.

The complications caused by uncontrolled blood glucose levels include dyslipidemia and pathological damage to organs, particularly the liver and kidneys. To assess these risks, we measured the levels of total cholesterol (TC), triglycerides (TG), high‐density lipoprotein cholesterol (HDL‐C) and low‐density lipoprotein cholesterol (LDL‐C) in the serum. As shown in **Figure** **5**a–d, Ins@Alg/NG had excellent regulatory effects on these serum lipids in diabetic mice. The study found that Ins@Alg/NG intervention reduced oxidative stress in diabetic mice induced by HFD and STZ, as confirmed by serum oxidative indexes including MDA, SOD, GSH, and CAT tests (Figure 5e–h). Additionally, the study showed that Ins@Alg/NG improved renal function in diabetic mice, as evidenced by lower levels of CRE and BUN (Figure 5i–j). According to the ELISA results, Ins@Alg/NG exhibited superior anti‐inflammatory properties in comparison to other groups, as indicated by lower levels of tumor necrosis factor (TNF‐α) and interleukin‐1β (IL‐1β) (Figure 5k,l).

### 2.8 Promotion of Insulin Sensitivity and Glucose Uptake by Ins@Alg/NG

**Figure 6:**
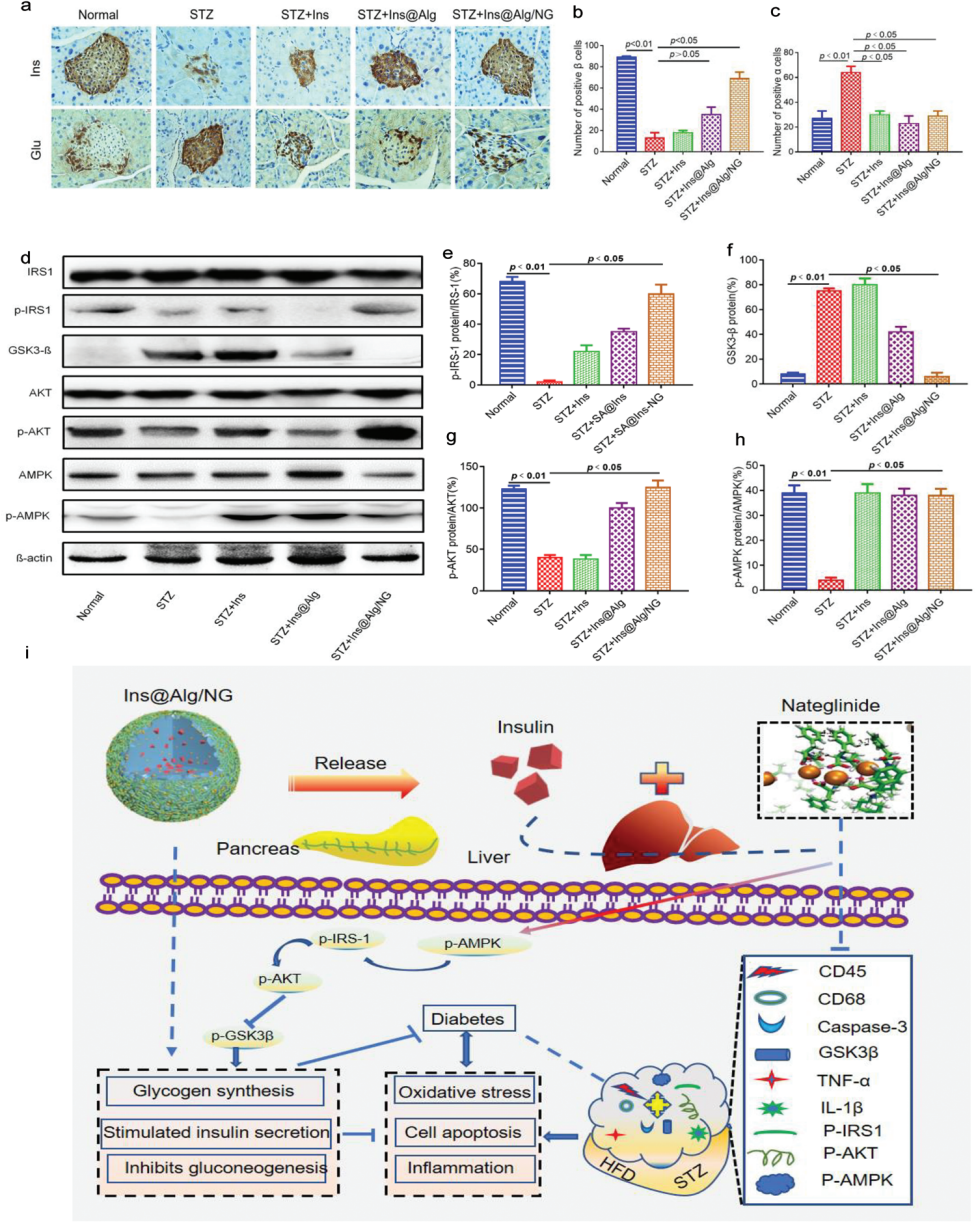
Ins@Alg/NG protected pancreas and liver from the diabetic injury. a) Insulin (Ins) and glucagon (Glu) were detected using IHC staining. b) Number of positive β cells. c) Number of positive α cells. d) Expressions of p‐IRS1, GSK3‐β, p‐AKT, and p‐AMPK in liver were detected by western blot. e–h) protein expression levels of p‐IRS1, GSK3‐β, p‐AKT, and p‐AMPK. i) Schematic illustration of anti‐diabetic molecular mechanism of Ins@Alg/NG. Data were presented as mean ± s.e.m (n = 3). Statistical significances were assessed using one‐way ANOVA with Bonferroni's post‐test. *p* < 0.05 or 0.01 was recognized as significant.

Ins resistance is a driving factor in the development of typeIIdiabetes. The impaired tyrosine phosphorylation of Ins receptor substance‐1 (IRS‐1) and inactivation of protein kinase B (AKT) are considered as biomarkers of Ins resistance in diabetic pancreas.^[^ ^48^ ^]^ The liver plays a crucial role in regulating blood glucose levels by controlling glucose uptake, synthesis, and metabolism. However, the abnormal expression of AMP activated protein kinase (AMPK) and glycogen synthase kinase‐3β (GSK‐3β) in the liver can be linked to Ins resistance, specifically in regards to glycogen synthesis.^[^ ^49^ ^]^ In this study, we utilized western blotting to measure the expression levels of IRS1, GSK3‐β, AKT, and AMPK. The results from **Figure** **6**d showed that the expression of phosphorylated IRS1 (p‐IRS1), p‐AKT and p‐AMPK proteins were significantly increased, while the expression of GSK3‐β protein was significantly decreased in the Ins@Alg/NG‐treated mice compared with the diabetic group (Figure 6e–h). These results suggested that Ins@Alg/NG ameliorated the Ins resistance, promoted glycogen synthesis and protected islet function mainly through activating IRS1, AKT, and AMPK expression while inhibited GSK3‐β expression (as illustrated in Figure 6i).

### 2.9 Biocompatibility and Safety of Ins@Alg/NG

In this study, we further conducted in vitro examination of the biocompatibility of Ins@Alg/NG. We used human normal liver cells (L‐02 cells) to test cell viability through MTT assay. Our findings indicate that there was no significant cytotoxicity observed on the L‐02 cells after incubated with Ins@Alg/NG (refer to Figure S12a, in the Supporting Information). To assess the restoring capability of Ins@Alg/NG on cell viability, a concentration of 35 µM palmitic acid (PA) was applied to induce cell lipotoxicity (Figure S12b, Supporting Information). This led to a significant raise in apoptotic protein Caspase‐3 expression and triggered cell apoptosis (Figure S13a,b, Supporting Information). However, Ins@Alg/NG significantly reversed the apoptosis and increased the viability of PA‐treated cells. We next studied the hemolysis rates of Ins@Alg/NG (Figure S14, Supporting Information), demonstrating that Ins@Alg/NG with the hemolysis rates all lower than 5% was proved as biocompatible in blood (Figure S14, Supporting Information). The biosafety of Ins@Alg/NG was also assessed in vivo through examining the pathological changes of main organs including liver, kidney, and pancreas during these 120‐day diabetic experiments. The hematoxylin‐eosin (Figure S15, Supporting Information) and Masson (Figure S16, Supporting Information) staining of liver and kidney sections showed that Ins@Alg/NG group had the similar morphology with normal group. More importantly, Ins@Alg/NG was capable of reducing the proliferation of collagen fibers, inhibiting inflammation in the liver and kidney, and protecting islet morphology from damage induced by STZ in the pancreas (indicated by the black arrow). These findings provide strong evidences that Ins@Alg/NG was biocompatible and safe for the treatment of diabetic occurrence.

## 3 Conclusion

The major finding in this study is that we demonstrate the helical assembly behavior of one short‐acting oral antidiabetic compound NG into left‐handed nanofibers, which is probably due to the existence of chiral phenylalanine residue on the backbone of the molecular structure. However, very different to the self‐assembly of pure phenylalanine‐based peptides as previously reported,^[^ ^50^ ^]^ the chiral assembly of NG occurs with aid of divalent cations under alkaline condition. At molecular level, MD simulations clearly revealed that ionic interaction between Ca^2+^ and deprotonated carboxylate anion of NG plays a vital role in maintaining the chiral assembly configuration accompanied by the interactions between benzene rings of NG. As far as we know, NG is likely the smallest phenylalanine derivative capable of assembling into helical nanofibers. These nanofibers have the potential to significantly improve short‐acting pharmacokinetic behavior and achieve long‐term action in vivo due to their prolonged retention and gradual degradation. With the merit of gastric acid resistance, NG nanofibers were designed to construct coating layer on the surface of Ca^2+^‐crosslinked alginate microgels to facilitate the oral Ins delivery. The Ins‐loading Alg/NG microgels Ins@Alg/NG were compared to free Ins and Ins‐encapsulating Alg microgels without NG coating. NG coating significantly prolonged the retention in gastrointestinal tract and specifically accumulated in liver organs over time, meanwhile pharmacokinetics of Ins@Alg/NG was markedly improved with greatest AUC compared to other groups, both indicating that the bioavailability of oral Ins using Ins@Alg/NG as carriers was enhanced (See Figure 4f for details). As expected, in vivo tests demonstrated that the sustained release and prolonged circulation of Ins and NG from Ins@Alg/NG synergistically regulated the blood glucose levels and restored the functions of damaged pancreatic islet functions in STZ‐induced diabetic mice after oral administration every 3 days. Additionally, Ins@Alg/NG improved Ins resistance by activating the expression of IRS1, AKT, and AMPK while repressing the level of GSK‐3β. The 90‐day tests conducted on Ins@Alg/NG showed no signs of toxicity and exhibited excellent biocompatibility when administered orally. This delivery system may also serve as a viable option for orally administering other antidiabetic drugs like Ins secretagogues agen,^[^ ^51^, ^52^, ^53^, ^54^ ^]^ glucagon‐like peptide‐1 receptor agonist^[^ ^55^ ^]^ and Ins analogues,^[^ ^56^ ^]^ which are commonly administered with NG in clinical applications.

## 4 Experimental Section

### Materials

Nateglinide (NG, purity ≥ 99%), insulin (Ins, purity ≥ 98%) and streptozotocin (STZ, purity ≥ 99.0%) were obtained from Shanghai Macklin Bio‐chem Co., Ltd (Shanghai, China). Palmitic acid (PA, purity ≥ 99.0%) and alginate (Alg, purity ≥ 90%, β‐D‐mannuronic/α‐L‐guluronic = 1:2) were obtained from Shanghai Aladdin Bio‐chem Technology Co., Ltd (Shanghai, China). The bicinchonininc acid (BCA) assay kit (PC0020) and caspase‐3 assay kit (C1115) were acquired from Beijing Soleibao Technology Co., Ltd (Beijing, China). The total cholesterol (TC) assay kit (A‐111‐1), triglyceride (TG) assay kit (A‐110‐1), high‐density liptein cholesterol (HDL‐C) assay kit (A112‐1‐1), low‐density lipoprotein cholesterol (LDL‐C) assay kit (A113‐1‐1), malondialdehyde (MDA) assay kit (A003‐1), superoxide dismutase (SOD) assay kit (WST‐1 method, A001‐3‐2), reduced glutathione (GSH) assay kit (A006‐2‐1), catalase (CAT) assay kit (A007‐1‐1), cyclization recombination enzyme (CRE) assay kit (C011‐2‐1), blood urea nitrogen (BUN) assay kit (C013‐2‐1) and tumor necrosis factor (TNF‐α) assay kit (H052) were purchased from Nanjing Jiancheng Bioengineering Institute (Nanjing, China). The interleukin‐1β (IL‐1β) assay kit (ml063132) were obtained from Shanghai Enzyme‐linked Biotechnology Co., Ltd (Shanghai, China). Hematoxylin and Eosin (H&E), terminal dexynucleotidyl transferase (TdT)‐mediated dUTP nick end labeling (TUNEL), Masson's trichrome staining (Masson), immunohistochemistry (IHC) and immunofluorescence (IFC) were obtained from Servicebio Co., Ltd (Wuhan, China). The primary antibodies were as follows: p‐IRS1 (1:500, Abcam, UK), IRS1 (1:500, Abcam, UK), GSK3‐β (1:500, Abcam, UK), p‐AKT (1:1000, Abcam, UK), AKT (1:1000, Abcam, UK), p‐AMPK (1:500, Abcam, UK), AMPK (1:500, Abcam, UK), β‐actin (1:200, Beyotime, China).

### Preparation of NG Gel

NG of 250 mg was completely dissolved in 10 mL solution (0.1 mol L^−1^ NaOH) at 60 °C for 1 h. Then, 10 mL CaCl2 (2 wt %) was added into NG solution, ultrasound 60 s, NG gel was formed after the temperature decreased to room temperature.

### Preparation of Ins@Alg/NG Microgels

Alginate aqueous solution (1.5 wt %) containing Ins (1 wt %) was sprayed into CaCl2 solution (2 wt %) by electro spraying technology to form Alg@Ins microgels (16 kV, 0.26 mL min^−1^). Then the microgels were placed in NG solution (25 mg mL^−1^) for ultrasound 60 s to obtain Ins@Alg/NG microgels.

### Microstructure Observation of NG Gel

The morphology of NG gel was observed by transmission electron microscopy (TEM, JEM‐2100, Hitachi, Tokyo, Japan). The sample solution was evenly dropped onto a copper grid, stained with 2% phosphotungstic acid and air‐dried at room temperature for TEM observation immediately at 100 kV. Further, scanning electron microscope (SEM, JSM‐7800F, Hitachi, Tokyo, Japan) and atomic force microscopy (AFM‐5500 M, Hitachi, Tokyo, Japan) were applied to observe the microstructure of NG gel. The structural characteristics of samples were scanned by Fourier transform infrared (FT‐IR) spectrometer (Perkin Elmer, Waltham, Ma, USA) in the range of 4000–400 cm^−1^. Circular dichroism (CD, J‐1500, JASCO, Tokyo, Japan) was used for secondary structure analysis of the sample. All scans were performed in the range of 200–400 nm with an average of 3 scans across all spectra.

### SAXS Measurements

The small‐angle X‐ray scattering (SAXS) measurements were carried out with the compact PILATUS camera, equipped with the SAXS/WAXS SYSTEM optical system of XENOCS (France). The Cu target X‐ray tube, reacted time as 900 s, operated at: U = 50 kV, I = 0.6 mA was used as a radiation source (λ = 0.1548 nm). The SAXS data were collected as a function of the scattering vector q = (4π/λ) sin θ, where 2θ was the scattering angle.^[^ ^57^, ^58^ ^]^

(1) $$\ln I\left(q\right)=\ln I\left(0\right)-\frac{1}{3}R_{G}^{2}q^{2}$$

(2) 2dsinθ=nλ

(3) d=2π/q

### Molecular Dynamics (MD) Simulations

All MD simulations and analyses were performed using the Gromacs‐2022. The charge parameters of deprotonated NGs were optimized by RESP2 based on Multiwfn.^[^ ^59^ ^]^ The Amber14sb_parmbsc1 all‐atom (AA) force field and Martini coarse‐grained (CG) force field were used in this study.^[^ ^60^, ^61^ ^]^ For the AA MD simulation, the initial condition was generated in a box of 8 × 8 × 8 nm^3^, which contained with 90 deprotonated NGs, 60 calcium ions (Ca^2+^), 30 chloride ions (Cl^−^) and 15285 water molecules. The temperature was coupled to 298 K using the V‐rescale method and the compressibility was coupled to 4.5e^−5^ (3e^−4^ in CG MD) using the Parrinello–Rahman method. The cutoff scheme of 1.0 nm (1.1 nm in CG MD) was implemented for the non‐bonded interactions. The covalent bonds were constrained with hydrogen atoms. The system was simulated for 200 ns in final production to ensure convergence. For the coarse‐grained (CG) MD simulation, the atom‐to‐bead mapping of deprotonated NGs followed the small molecules mapping standard, and the bonded parameters were generated using Bartender.^[^ ^62^ ^]^ The initial condition was generated in a box of 40 × 40 × 40 nm^3^, which contained with 7292 deprotonated NGs, 3646 calcium ions and 51633 CG water beads (corresponding to 206532 water molecules). The system was simulated for 1 µs in final production to ensure convergence. Image rendering was performed using VMD 1.9.3.

### Release Profile in Simulated Gastrointestinal Fluids

Ins@Alg/NG was placed into the simulated gastric juice (SGF), simulated intestinal fluid (SIF) and simulated intestinal fluid (SCF) at 37 °C incubator, respectively. At predetermined time points (6, 12, 24, and 48 h), a 2 mL sample of liquid was extracted using 5 mL of methanol through ultrasonic extraction for 30 min, and then filtered. The degradation behavior of Ins@Alg/NG in the gastrointestinal environment was assessed by determining the content of free Ins and NG using high‐performance liquid chromatography (HPLC).

### In Vivo Distribution of Ins@Alg/NG Microgels

To evaluate the biodistribution of Ins@Alg/NG microgels in vivo, Cy7 was used as a fluorescent probe in Ins@Alg/NG microgels instead of Ins. Then, diabetic mice were administrated with free Cy7, Cy7@Alg, or Cy7@Alg/NG (Cy7 at 1 mg kg^−1^). The changes of fluorescent intensity in mice were observed at the predetermined time points (3, 6, 12, 24, 48, and 72 h) by an in vivo imaging system IVIS (PerkinElmer).

### In Vivo Anti‐Diabetic Experiments

Eight‐weeks old Balb/c female mice (weight 25 ± 3 g) were purchased from Changsheng Biological Co., Ltd with a Certificate of Quality No. SYXK (Liao) 2017‐0005 (Dalian, China). The animal experiments were conducted in accordance with the Guide for the Care and Use of Laboratory Animals and the Regulations of Experimental Animal Administration issued by the Ethical Committee for Laboratory Animals at Dalian Polytechnic University (Permit No.: 201 726 221 101 893 184). The experiments were performed with strict adherence to ethical standards.

To construct the diabetic model, mice were adaptively housed for 7 days and then fed with a high fat diet for 10 days, with the exception of the blank group. Intraperitoneal injections of STZ (50 mg kg^−1^) were administered six times, with a 5‐day interval between each injection. Diabetic mice were identified if blood sugar remained stable above 16.7 mmol L^−1^ for 10 days and used for the following experiments. Diabetic mice were randomly divided into 5 groups (n = 10), namely, blank normal group (receiving untreated drinking water), STZ group (STZ, 50 mg kg^−1^), STZ+Ins group (Ins, 20 UI kg^−1^), STZ+Ins@Alg group (Ins@Alg, 150 mg kg^−1^ containing Ins 20 IU kg^−1^) and STZ+Ins@Alg/NG group (Ins@Alg/NG, 150 mg kg^−1^ containing Ins 20 IU kg^−1^). Each group was orally gavaged by these samples every 3 days. Through the whole experiment, the body weight, diet, drinking water and blood sugar of mice were monitored every day. After 130 days of sample intervention, the mice were sacrificed, and the blood was collected for the subsequent analysis.

Tissue sections were stained using immunohistochemistry and immunofluorescence analysis. The liver, kidney, and pancreas tissues were fixed in 4% paraformaldehyde, embedded in paraffin, and cut into 5 µm thick sections. All sections were placed in xylene (15 min), and then dewaxed with 95%, 75%, and 50% ethanol (15 min). Stained with hematoxylin‐eosin (H&E) and Masson trichrome according to the protocol. Immunohistochemical (IHC) staining was performed according to the manufacturer's protocols (Boster, China). Pancreas sections were incubated with anti‐insulin mouse mAb (Servicebio, GB11097) and anti‐glucagon mouse mAb (Servicebio, GB12097) at 4 °C overnight. The sections were then washed and incubated with horseradish peroxidase (HRP) conjugated goat anti‐mouse IgG (Servicebio, GB23301). Slides were covered and images were observed and collected by a fluorescence microscope (Nikon, Eclipse C1, Japan).

For immunohistofluorescence, pancreas sections were incubated with anti‐insulin mouse mAb (Servicebio, GB11097) at 37 °C for 0.5 h, then anti‐glucagon mouse mAb (Servicebio, GB12097) at 4 °C overnight, anti‐CD45 mouse mAb (Servicebio, GB14038) and anti‐CD68 mouse mAb (Servicebio, GB14043) were added at 4 °C overnight, respectively. Pancreas tissue incubated with FITC conjugated goat anti‐mouse IgG (Servicebio, GB22301) in dark for. 1.5 h. Finally, Pancreas tissue were counterstained with 4′,6‐diamidino‐2‐phenylindole (DAPI, Servicebio, GDP1024) for the nuclei. Slides were covered and images were observed and collected by a fluorescence microscope (Nikon, Eclipse C1, Japan).

In this study, biochemical parameters were assessed to evaluate the impact of oxidative stress on kidney tissues. Blood lipid indexes such as TC, TG, LDL‐C, and HDL‐C were measured from the serum, while renal function was evaluated using serum CRE and BUN with commercial kits. Oxidative stress markers including CAT, GSH, SOD, and MDA were also detected using commercially available assay kits. Additionally, the concentrations of inflammatory factors IL‐1β and TNF‐α in the serum were determined using an enzyme‐linked immunosorbent assay kit. All procedures were strictly conducted in accordance with the instructions provided with the kit.

Western blotting analysis was performed by extracting liver histamine using RIPA lysis buffer and quantifying it using bicinchoninic acid. An equal amount of protein (50 µg lane^−1^) was separated on a 12% SDS‐PAGE gel and transferred to a polyvinylidene fluoride (PVDF) membrane. Then, the membrane was sealed with 3% BSA for 1.5 h in tris‐buffered saline (TBS) containing 0.05% Tween‐20 at room temperature. After that, samples were incubated overnight with primary antibody at 4 °C. Subsequently, the membrane was subjected to three washes with tris‐buffered saline tween (TBST) for three times, each time for 8 min, followed by incubation with a secondary antibody at room temperature for 60 min. Finally, the resulting single protein band was then examined using emitter coupled logic (ECL) substrate (Pierce Chemical Co., Rockford, IL, USA). The proteins were visualized by a chemiluminescence detection system (ChemiDoc Touch, Bio‐Rad, USA). Band intensity was analyzed with Image Plus 6.0 software.

### Statistical Analysis

All data were presented as mean ± s.e.m. Statistical significances were assessed using one‐way ANOVA with Bonferroni's post‐test. *p* < 0.05 or 0.01 was recognized as significant.

## Conflict of Interest

The authors declare no conflict of interest.

## Author Contributions

Y.F.L., L.H.C., and J.N.H. conceived and designed the experiments. Y.X. and D.W. helped with characterization and animal experiments. S.H.L. contributed to the mouse experiment. H.J.Y., R.N.Z., and T.C. performed the cell toxicity and live/dead experiment, and histological staining. Y.F.L. analyzed the data and helped revise the paper and developed the concept and supervised the study. Y.F.L and L.H.C. wrote the manuscript.

## Supporting information

Supporting Information

Supplemental Movie 1

## Data Availability

The data that support the findings of this study are available from the corresponding author upon reasonable request.
