# Supplementary material for: Helical‐Like Assembly of Nateglinide as Coating for Oral Delivery of Insulin and Their Synergistic Prevention of Diabetes Mellitus
Source: Adv Sci (Weinh). 2023 Aug 16;10(29):2301879. doi: 10.1002/advs.202301879 (PMC10582466; doi:10.1002/advs.202301879)
Supplement: Supplementary file 1 — Supporting Information [file ADVS-10-2301879-s001.pdf]

ADVANCED SCIENCE
Open Access

Supporting Information

for *Adv. Sci.*, DOI 10.1002/advs.202301879

Helical-Like Assembly of Nateglinide as Coating for Oral Delivery of Insulin and Their Synergistic Prevention of Diabetes Mellitus

*Yanfei Li, Lihang Chen, Yu Xu, Sihui Li, Huijia Yan, Tao Chen, Ziqi Hua, Di Wu, Runan Zhao and Jiangning Hu**

Helical-like assembly of nateglinide as coating for oral delivery of insulin and their synergistic prevention of diabetes mellitus

*Yanfei Li [1], Lihang Chen[1], Yu Xu[1], Sihui Li[1], Huijia Yan[1], Tao Chen[1], Ziqi Hua[1], Di Wu[1], Runan Zhao[2], Jiangning Hu [1] **

[1] SKL of Marine Food Processing & Safety Control, National Engineering Research Center of Seafood, Collaborative Innovation Center of Seafood Deep Processing, School of Food Science and Technology, Dalian Polytechnic University, Dalian 116034, China

🖂 **To the Correspondence**

Name: Jiang-Ning Hu

🖂 E-mail: hujiangning2005@hotmail.com

Mailing address: SKL of Marine Food Processing & Safety Control, National Engineering Research Center of Seafood, Collaborative Innovation Center of Seafood Deep Processing, School of Food Science and Technology, Dalian Polytechnic University, Dalian 116034, China

Telephone: +86-411-86318731

Supplement information materials

Encapsulation efficiency and drug loading of Ins and NG: Microspheres were

crushed and then dissolved in methanol, 0.45 μm microporous membrane was used for ultrasonic filtration. the content of Ins and NG were determined by 1260 high performance liquid chromatography (HPLC) (Agilent Technologies Inc., California, USA). For Ins, the detection wavelength was 214 nm, the mobile phase consisted of a mixture of acetonitrile and ultrapure water (containing 0.1% phosphate) (30: 70, v/v), flow rate was 0.8 mL/min and retention time was 15 min. For NG, the detection wavelength was 210 nm, the mobile phase consisted of a mixture of acetonitrile and ultrapure water (containing 0.1% phosphate) (54: 46, v/v), flow rate was 0.8 mL/min and retention time was 15 min. Based on the standard concentration and peak area, the sample content was calculated. Encapsulation ratio (EE) and drug loading (DL) were calculated as follows: [1]

$$\text{EE}\ (\%) = \frac{\text{The weight of Ins/NG in microspheres}}{\text{The weight of initial Ins/NG}} \times 100\% \qquad (1)$$

$$\text{DL}\ (\%) = \frac{\text{The weight of Ins/NG in microspheres}}{\text{The weight of microspheres}} \times 100\% \qquad (2)$$

Rheological testing: The rheological properties of each sample were measured by the rheometer (DHR-1, TA, USA). The prepared gel was equilibrated at room temperature for 2 hours. PP25 plate was selected with a gap of 1 mm, and rheological measurement was performed at 25 °C. To test the oscillatory shear rheology, appropriate amount of NG gel was used and the dynamic sweep frequency was measured at 0.01% strain, and the frequency was between 0.01 Hz and 100 Hz. The rheological behavior of NG gel was studied by temperature scanning test between 25 °C to 65 °C, at 10 Hz. Dynamic frequency scanning was used to further detect the viscoelastic properties of the gel, and the linear viscoelastic region was selected from the angular frequency range of 0.1 to

100 rad/s.

Hemolysis analysis: Blood samples from mice were added to NaCl solution and were centrifuged at 1500 rpm for 10 min to obtain red blood cells (RBCs). After washed with NaCl solution, the RBCs were diluted to 1/50 of their volume with PBS solution (pH 7.2). The samples were divided into following groups: (1) Positive control group: purified water; (2) Negative control group: PBS; (3) Ins@Alg/NG: 0.5 mg/mL; (4) Ins@Alg/NG: 1.0 mg/mL; (5) Ins@Alg/NG: 1.5 mg/mL. The diluted red blood cell suspension was mixed evenly with the above samples and incubated at 37 °C for 3 hours. After centrifugation (1500 rpm for 10 min) at room temperature, the absorbance of the supernatant at 541 nm was determined by microplate analyzer.

Particle size analysis of Ins@Alg and Ins@Alg/NG: Microspheres were observed by light microscopy (Leica DM2500, Leica Microsystems GmbH, Wetzlar, Germany). Particle size analysis of microspheres (n>100) were performed using Image J software and standard deviation calculated.

Cytotoxicity testing of Ins@Alg and Ins@Alg/NG: The cytotoxicity of Ins@Alg and Ins@Alg/NG to human normal liver cells (L-02) was determined. L-02 cells were inoculated in 96-well plates (density 10^5 μL/ well) and cultured in an incubator for 24 hours. After cell adhesion, the cells were treated with different concentrations of Ins@Alg and Ins@Alg/NG (0, 5, 10 and 20 μmol/mL). After treated for 24 hours, MTT (3-(4,5-dimethylthiazol-2-yl)-2,5-diphenyltetrazolium bromide) was added and the mixtures were incubated at 37 °C for 4 hours. The culture medium was removed and 200 μL DMSO was added into each well, and the absorbance was determined at 490

nm using a microplate reader.

Cytotoxicity testing of PA: The cytotoxicity of palmitic acid (PA) to L-02 cells was determined. L-02 cells were inoculated in 96-well plates (density 10^5 μL/ well) and cultured in an incubator for 24 hours. After cell adhesion, the cells were treated with different concentrations of PA (0, 5, 10 ,15, 20, 25, 30, 35 and 40 μmol/mL) for 24 hours. After treated for 24 hours, MTT was added and the mixtures were incubated at 37 °C for 4 hours. The culture medium was removed and 200 μL DMSO was added into each well, and the absorbance was determined at 490 nm using a microplate reader.

Ins@Alg/NG increases glucose consumption by L-02 cells: L-02 cells were inoculated in 12-well plates and cultured in incubators for 24 hours， then, cells were treated with PA (concentration of 30 μmol/mL). After cultured for 24 hours, Ins@Alg and Ins@Alg/NG (concentration of 20 μmol/mL) were added into the culture. At 24 and 48 hours, MTT was added and the mixtures were incubated at 37 °C for 4 hours. The culture medium was removed and 200 μL DMSO was added into each well, and the absorbance was determined at 490 nm using a microplate reader.

Apoptosis analysis of Ins@Alg/NG: The protective effect of Ins@Alg/NG on apoptosis of L-02 cells induced by PA was determined. L-02 cells were inoculated into 6-well plates (density 10^5 μL/ well) and cultured in an incubator for 24 hours. After cell adhesion, Ins@Alg and Ins@Alg/NG (20 μmol/mL) were added and incubated for 24 hours. After 24 hours, PA (30 mg/mL) was added, and the culture continued for 24 hours. The fluorescence staining was continued according to the instructions of the Caspase-3 kit, and incubated at 37 °C for 30 min. The expression of apoptotic cells was observed

under inverted fluorescence.

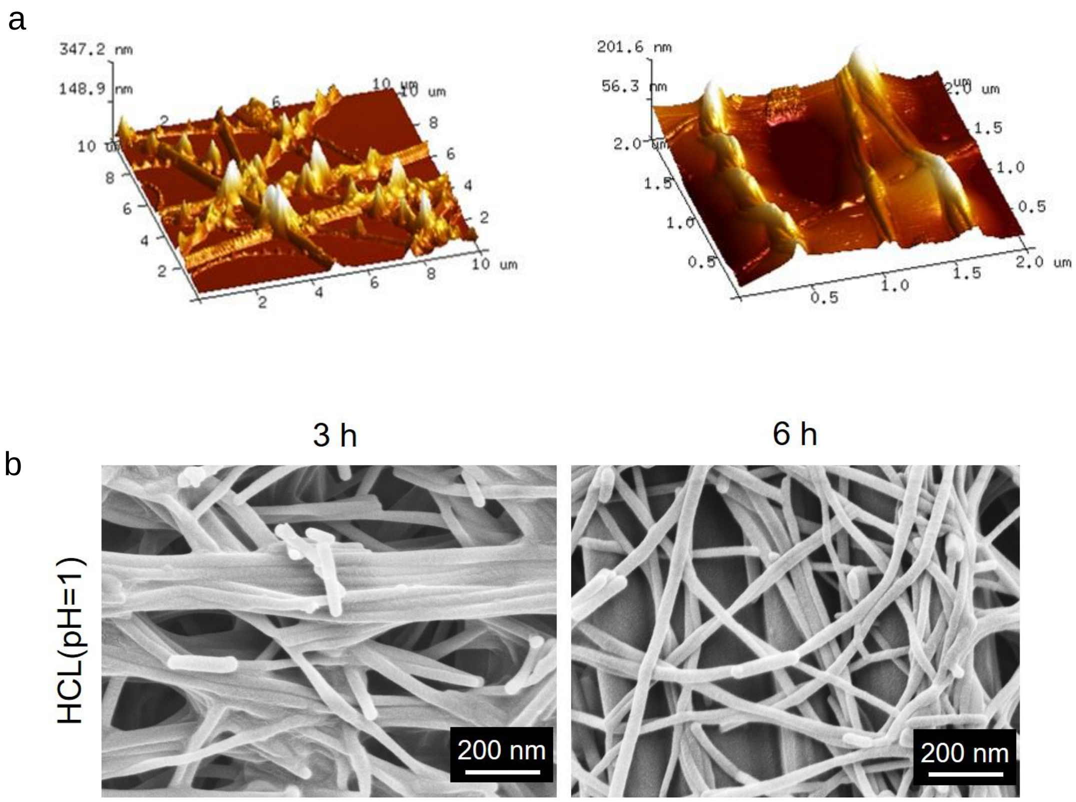

Figure S1. Characterization and stability of NG-Ca^{2+} gel. a) 3D drawing of AFM. b) 3 and 6 h after HCL (pH 1) was added to NG-Ca^{2+} gel, the microstructure changes were observed by SEM.

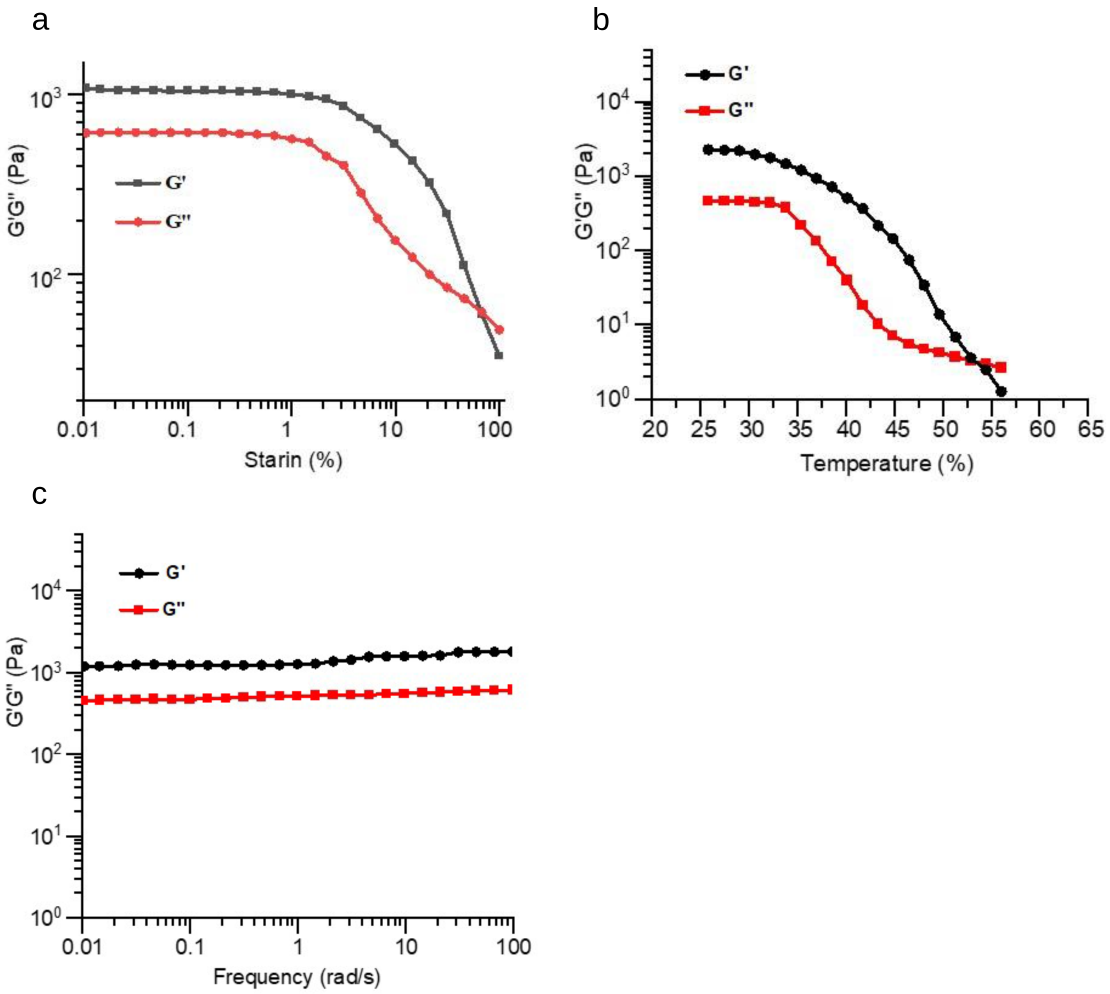

Figure S2. Rheological properties of the NG-Ca^{2+} gel. a) Strain-dependent oscillatory shear rheology of the NG-Ca^{2+} gel at a fixed frequency of 100 Hz. b) NG-Ca^{2+} gel responsiveness to temperature, fixed frequency (100 Hz) and strain (0.01%). c) Dynamic frequency sweeps of the NG-Ca^{2+} gel was measured at 0.01% strain.

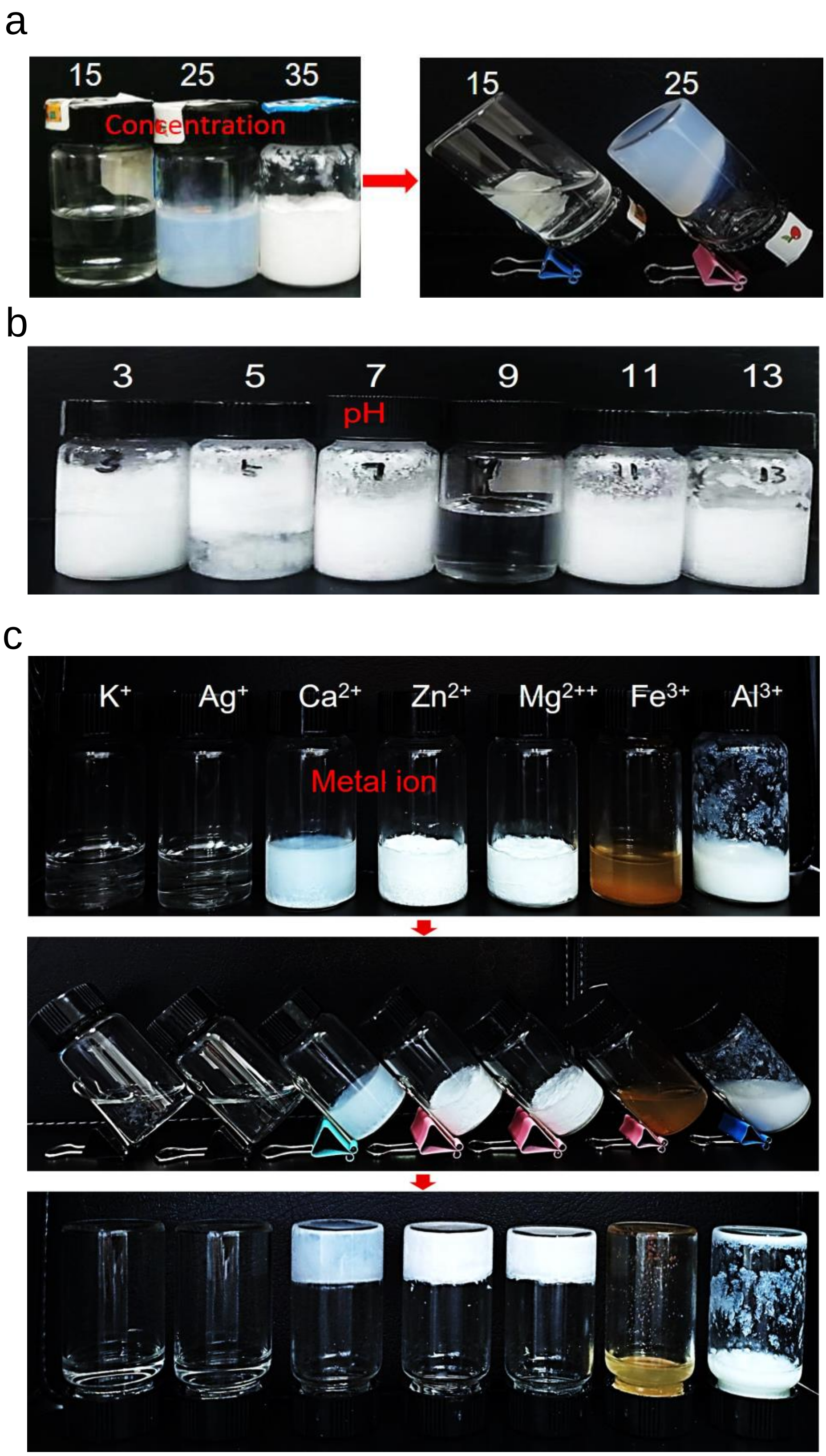

Figure S3. Test of NG gel formation conditions. a) NG gel diagram of different concentrations (15, 25 and 35 mg/mL). b) The influence of different pH conditions on

the formation of NG gel. c) Exploration of gels formed by different metal ions.

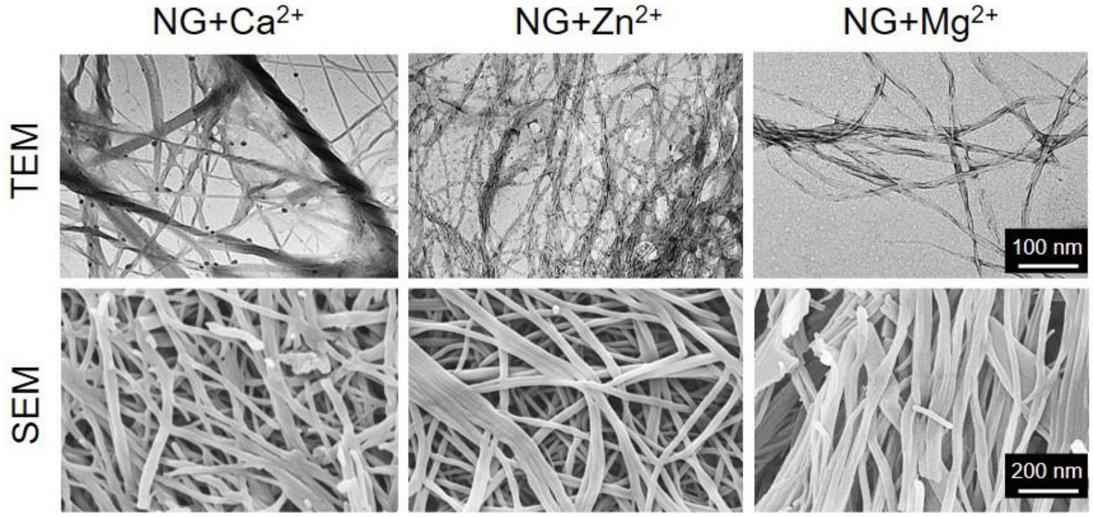

Figure S4. TEM and SEM of gel formation by Ca^{2+}, $Zn^{2+}$and Mg^{2+}.

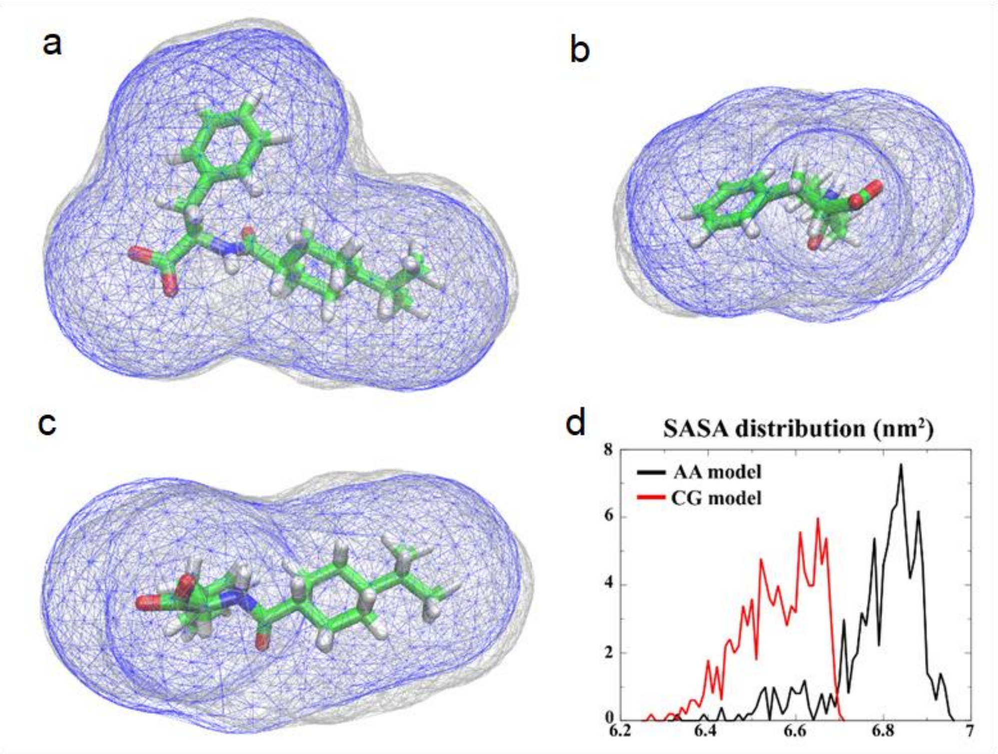

Figure S5. Overlap the plot of AA and CG Connolly surfaces of deprotonated NGs (AA is in gray, CG is in blue). a) Top view. b-c) Left view and front view. d) SASA distributions along the trajectory.

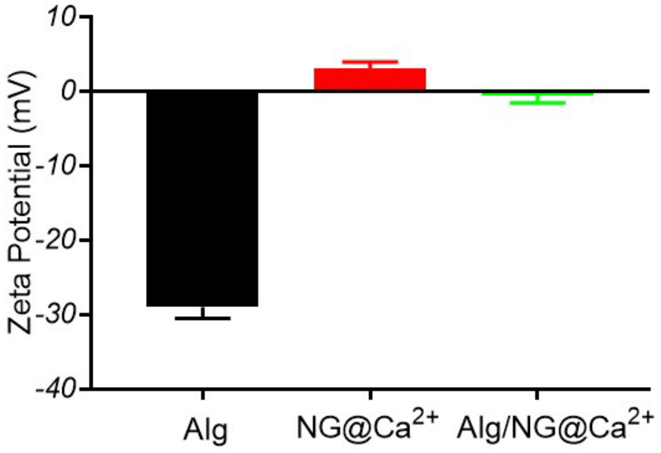

Figure S6. Zeta potential analysis of Ins@Alg/NG.

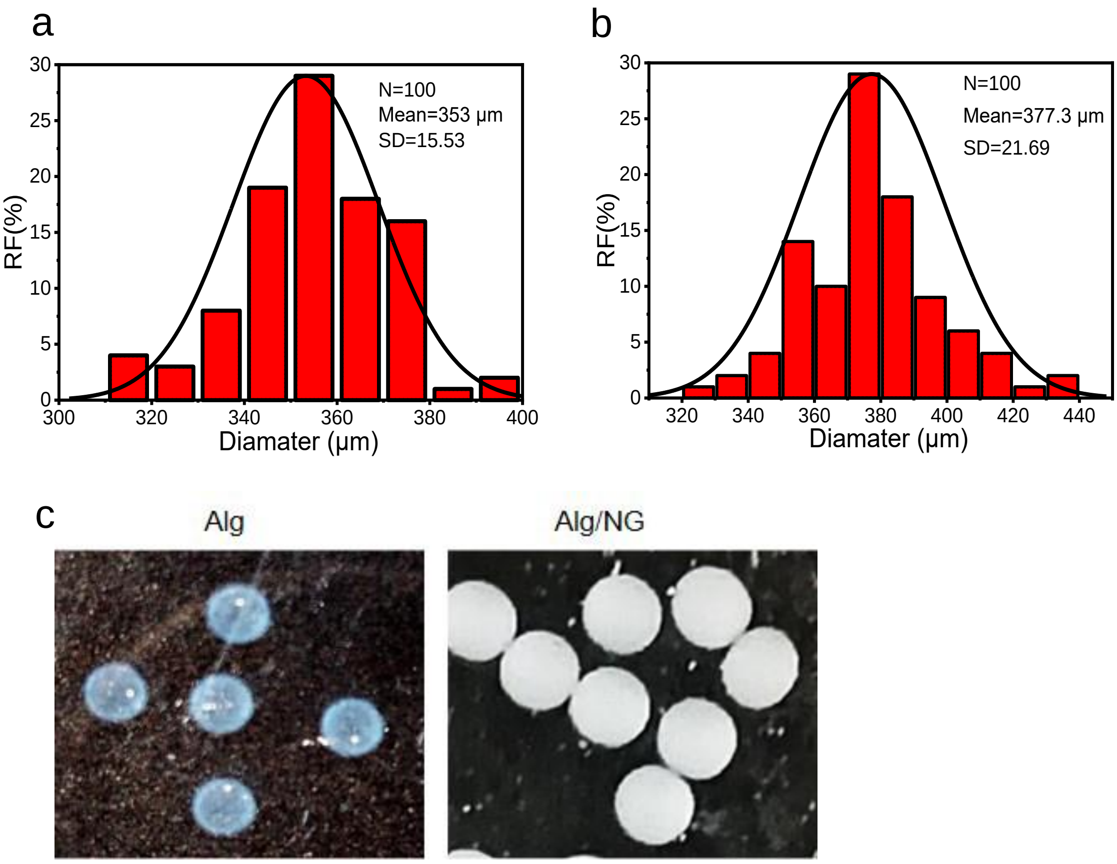

Figure S7. Preparation and characterization of Alg and Alg/NG. a,b) Particle size distribution of Alg and Alg/NG. c) Microscopic images of Alg and Alg/NG.

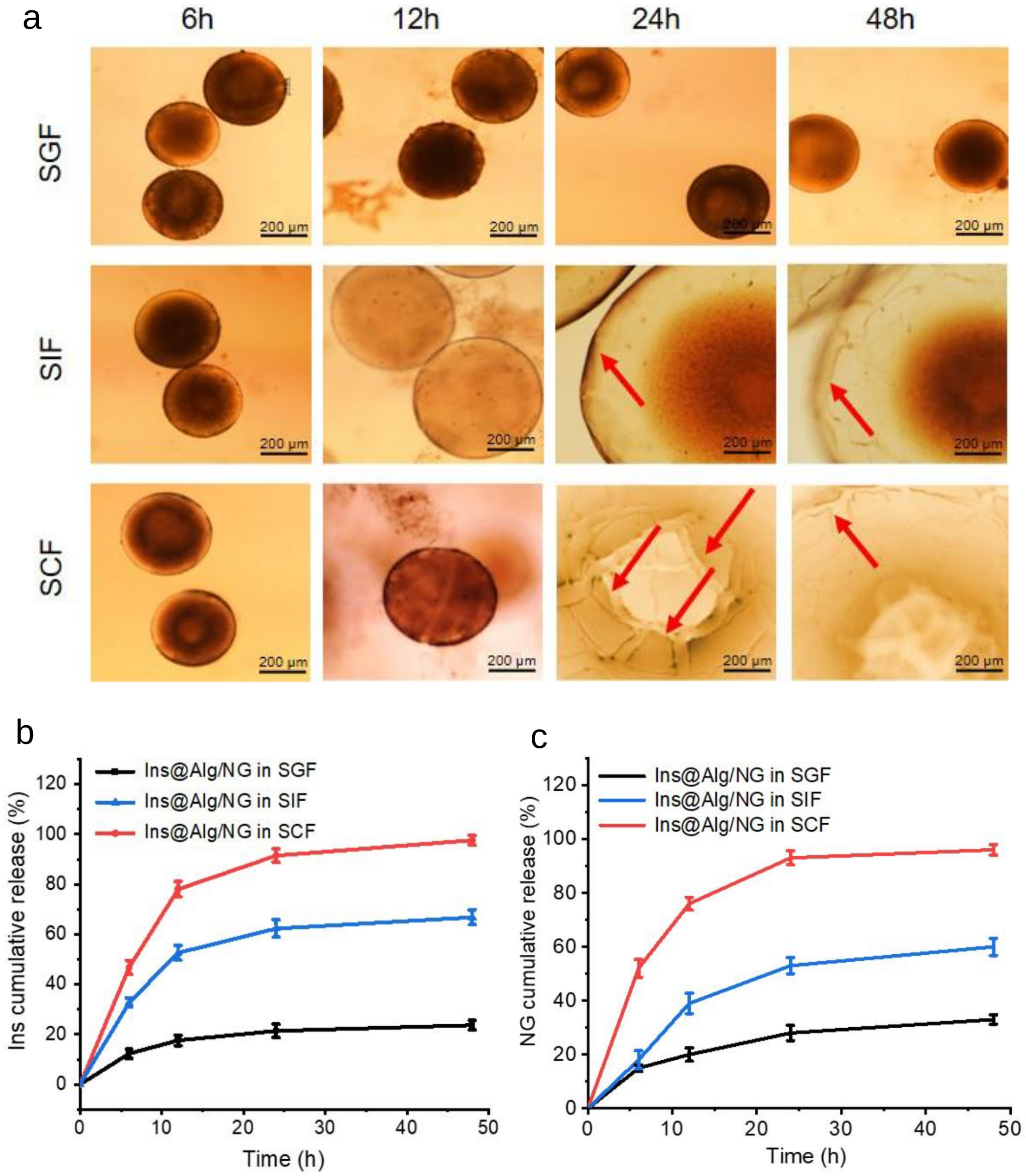

Figure S8. Stability of Ins@Alg/NG and simulated release of gastroenteric fluid. a) Microscope observation of artificial gastroenteric fluid at different time periods *in vitro* at Ins@Alg/NG. b) Release of Ins in gastroenteric fluid. c) Release of NG in gastroenteric fluid. Red arrow points to NG gel shell, Data were presented as mean ± s.e.m (n=3). Statistical significance was assessed using one-way ANOVA with Bonferroni's post-test.

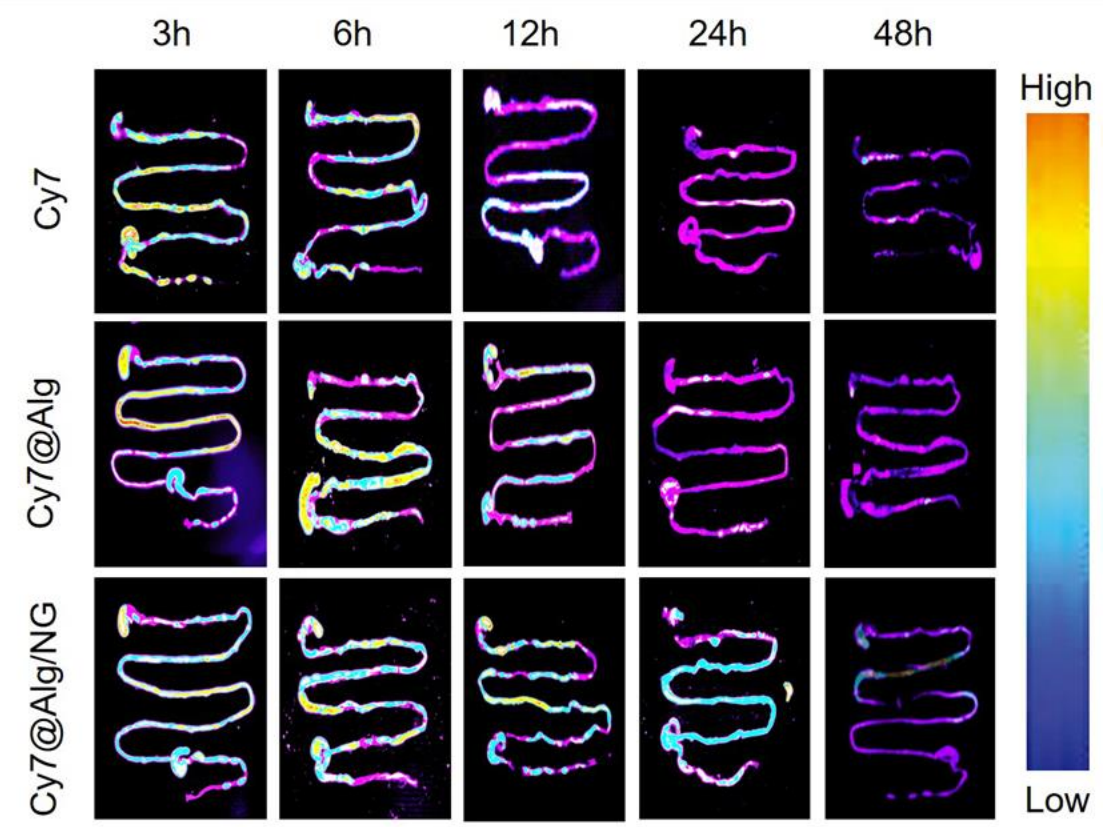

Figure S9. Distribution of Cy7, Cy7@Alg and Cy7@Alg/NG in intestinal tract at different time periods.

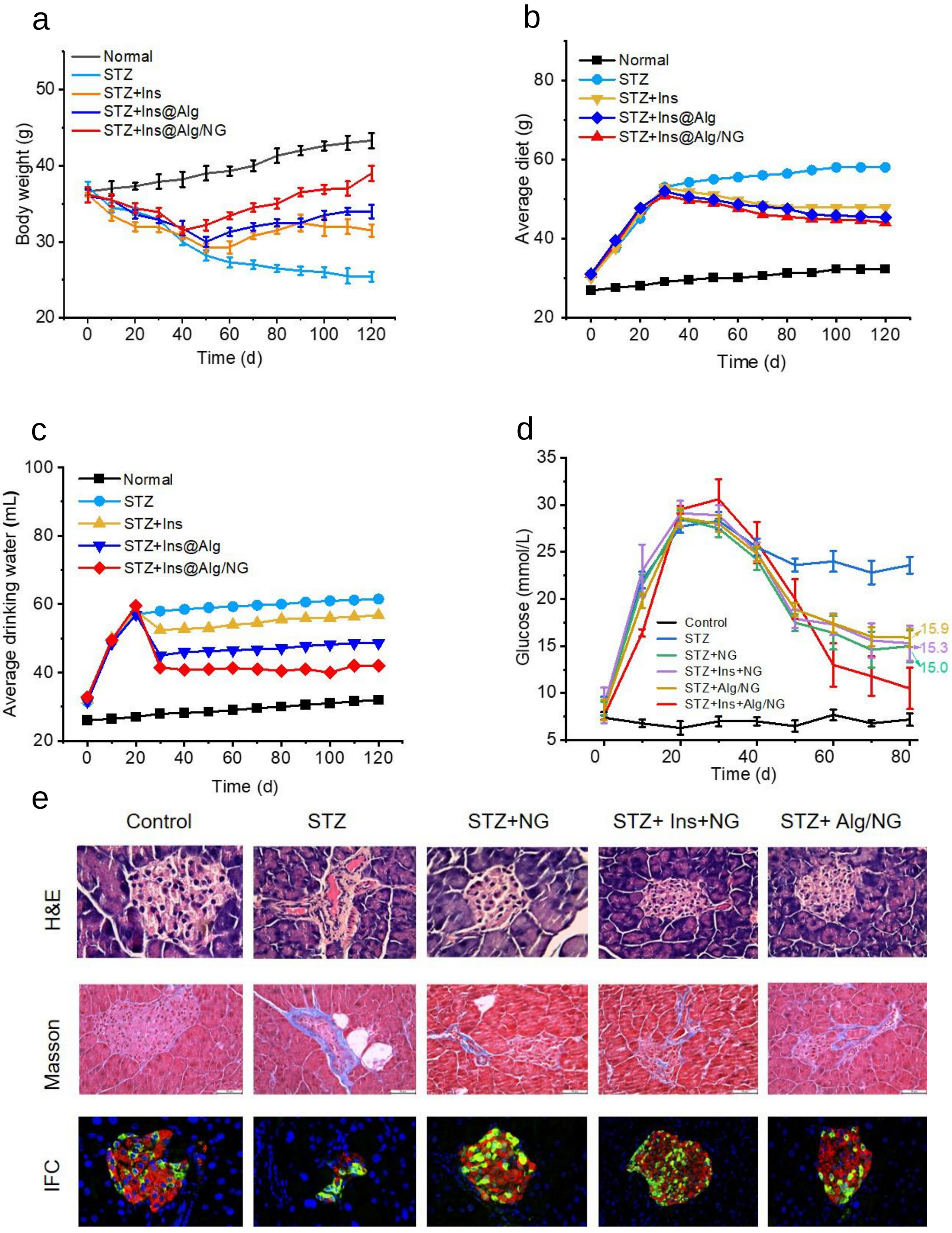

Figure S10. Ins@Alg/NG improved symptoms in diabetic mice. a) Basal body weight change. b) Dietary quantity. c) Water intake. d) Blood glucose levels of STZ+NG, STZ+Ins+NG and STZ+Alg/NG. e) H&E, Masson and immunofluorescence staining for STZ+NG, STZ+Ins+NG and STZ+Alg/NG. Data were presented as mean ± s.e.m (n=7-10). Statistical significance was assessed using one-way ANOVA with Bonferroni's post-test.

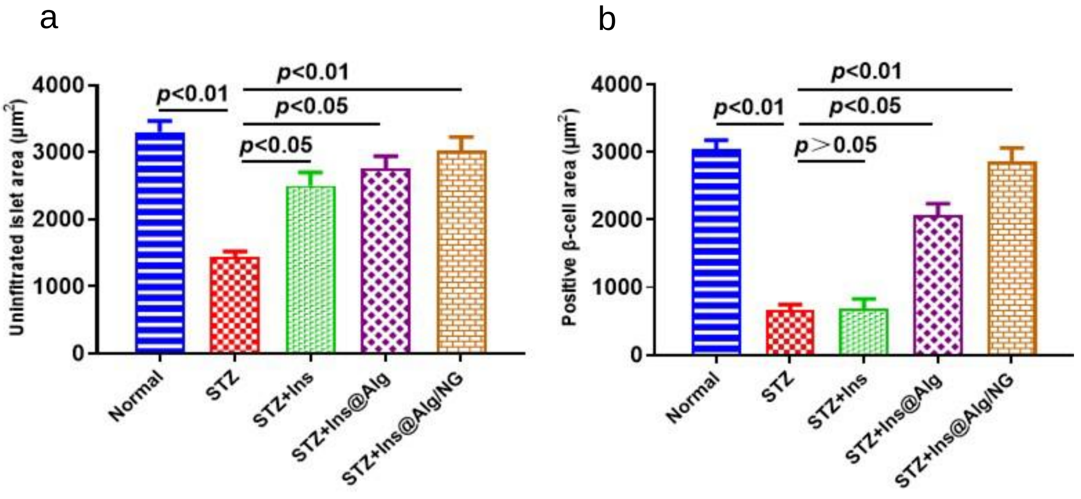

Figure S11. Ins@Alg/NG effectively protected pancreatic function. a) Uninfected islet area. b) Positive of β-cell area. Data were presented as mean ± s.e.m (n=3). Statistical significance was assessed using one-way ANOVA with Bonferroni's post-test.

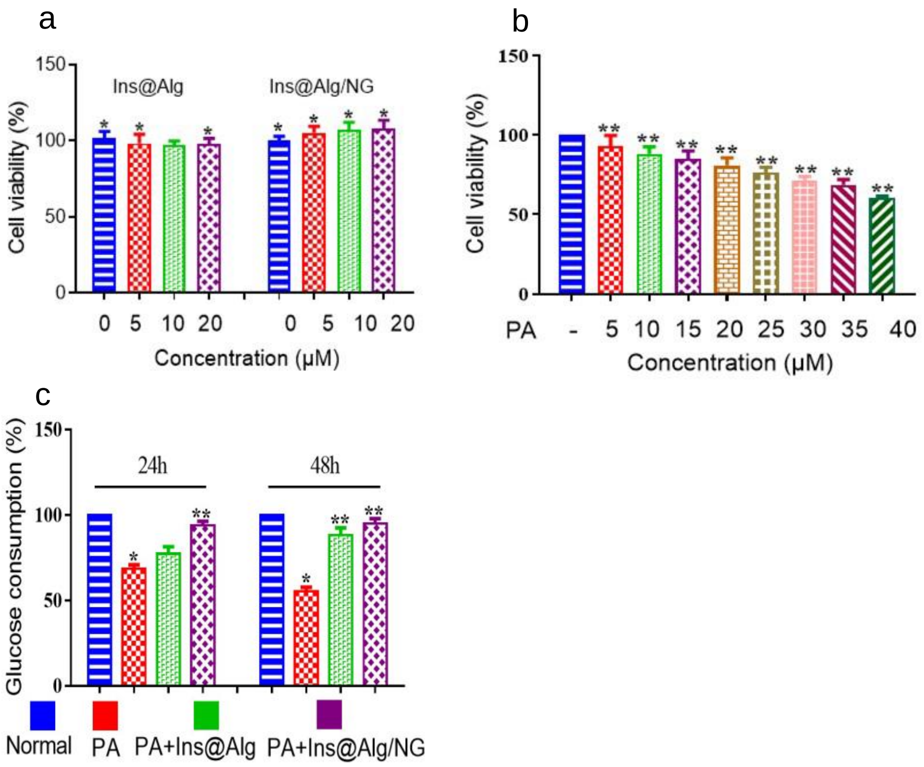

Figure S12. Ins@Alg/NG reversed PA-induced damage to L-02 cells. a) The viability of L-02 cells after treated with Ins@Alg and Ins@Alg/NG. b) The effect of PA on the viability of L-02 cells. c) Ins@Alg/NG increased glucose consumption in L-02 cells. Data were presented as mean ± s.e.m (n=7-10). Statistical significance was assessed using one-way ANOVA with Bonferroni's post-test.

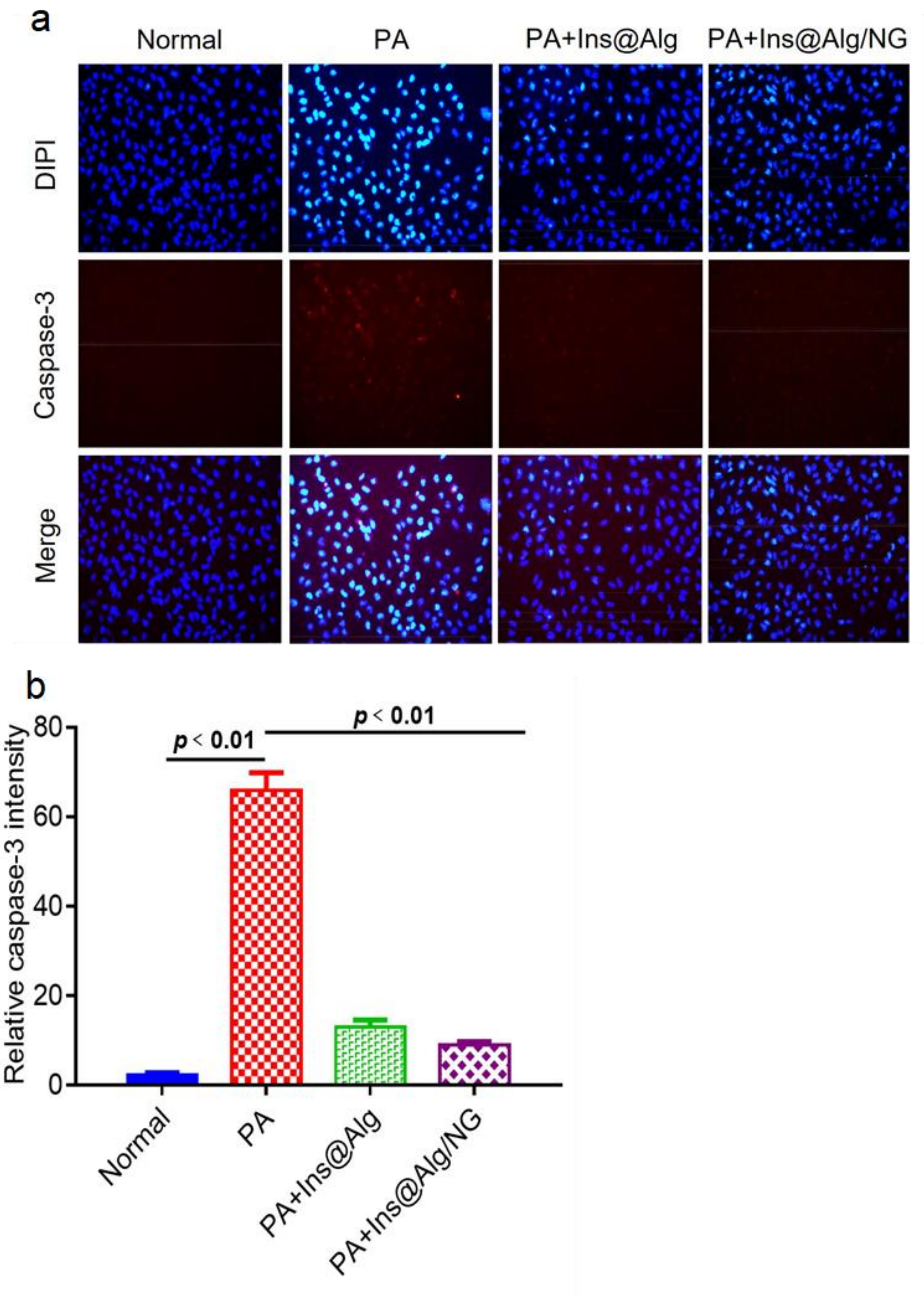

Figure S13. Immunofluorescence detection of PA-induced apoptosis of L-02 cells reversed by Ins@Alg/NG. a) Ins@Alg/NG improved PA-induced the apoptosis of L-02 cells. b) Positive cell expression. Data were presented as mean ± s.e.m (n=3). Statistical significance was assessed using one-way ANOVA with Bonferroni's post-test.

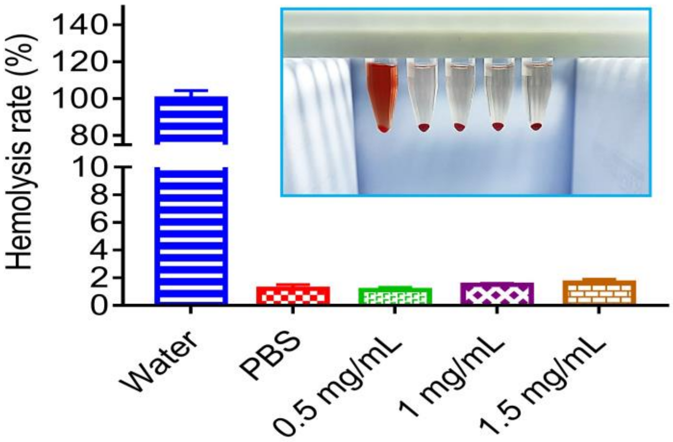

Figure S14. Biocompatibility test of Ins@Alg/NG. Data were presented as mean ± s.e.m (n=3). Statistical significance was assessed using one-way ANOVA with Bonferroni's post-test.

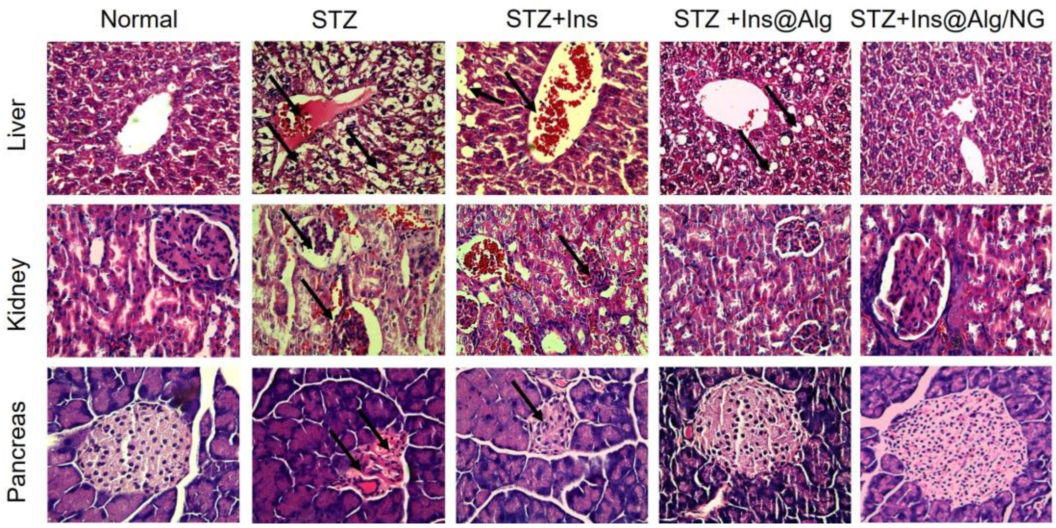

Figure S15. H&E staining of main organs after treated with Ins@Alg/NG in diabetic mice.

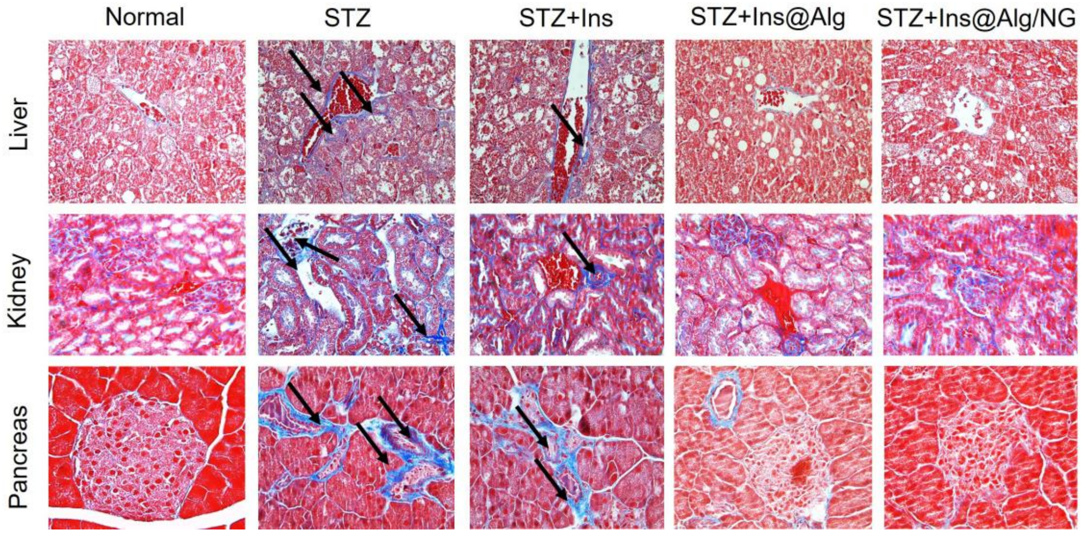

Figure S16. Masson staining of main organs after treated with Ins@Alg/NG in diabetic mice.

References

[1] X. Yu, L. Xiang, G. Wei, H. Hai-Bo, Z. Beiwei, H. Jiang-Ning, *J. Agric. Food Chem*. **2020**, 68, 8545.
